# Temporal Regulation of Lipin Activity Diverged to Account for Differences in Mitotic Programs

**DOI:** 10.1016/j.cub.2015.11.061

**Published:** 2016-01-25

**Authors:** Maria Makarova, Ying Gu, Jun-Song Chen, Janel Renée Beckley, Kathleen Louise Gould, Snezhana Oliferenko

**Affiliations:** 1 Randall Division of Cell and Molecular Biophysics, King’s College London, London SE1 1UL, UK; 2 Department of Cell and Developmental Biology, Vanderbilt University, Nashville, TN 37240, USA

## Abstract

Eukaryotes remodel the nucleus during mitosis using a variety of mechanisms that differ in the timing and the extent of nuclear envelope (NE) breakdown. Here, we probe the principles enabling this functional diversity by exploiting the natural divergence in NE management strategies between the related fission yeasts *Schizosaccharomyces pombe* and *Schizosaccharomyces japonicus* [1, 2, 3]. We show that inactivation of Ned1, the phosphatidic acid phosphatase of the lipin family, by CDK phosphorylation is both necessary and sufficient to promote NE expansion required for “closed” mitosis in *S. pombe*. In contrast, Ned1 is not regulated during division in *S. japonicus*, thus limiting membrane availability and necessitating NE breakage. Interspecies gene swaps result in phenotypically normal divisions with the *S. japonicus* lipin acquiring an *S. pombe*-like mitotic phosphorylation pattern. Our results provide experimental evidence for the mitotic regulation of phosphatidic acid flux and suggest that the regulatory networks governing lipin activity diverged in evolution to give rise to strikingly dissimilar mitotic programs.

## Highlights

• Lipin phosphorylation by CDK drives mitotic nuclear envelope expansion in *S. pombe*
• CDK-dependent lipin phosphorylation is required for closed mitosis
• Lipin is not regulated by mitotic CDK in the related species *S. japonicus*
• Interspecies gene swaps reveal species-specific trans-regulation of lipin activity

## Teaser

Using closely related yeasts, Makarova et al. uncover a molecular basis for variability in nuclear envelope expansion during mitosis. They show that cells undergoing closed mitosis expand their nuclear envelope prior to division by entraining inactivation of the phosphatidic acid flux regulator lipin to high CDK activity.

## Results and Discussion

The surface area of a mother nucleus undergoing closed mitosis must increase to allow intranuclear mitotic spindle elongation and formation of the daughter nuclei. The model yeasts *S. pombe* and *Saccharomyces cerevisiae* solve this problem through nuclear envelope (NE) expansion at mitotic entry [1, 4, 5, 6, 7, 8, 9]. In contrast, *S. japonicus*, an *S. pombe* relative, does not expand its NE and instead relies on NE breakdown during anaphase to allow chromosome segregation [1, 3].

**Figure 1:**
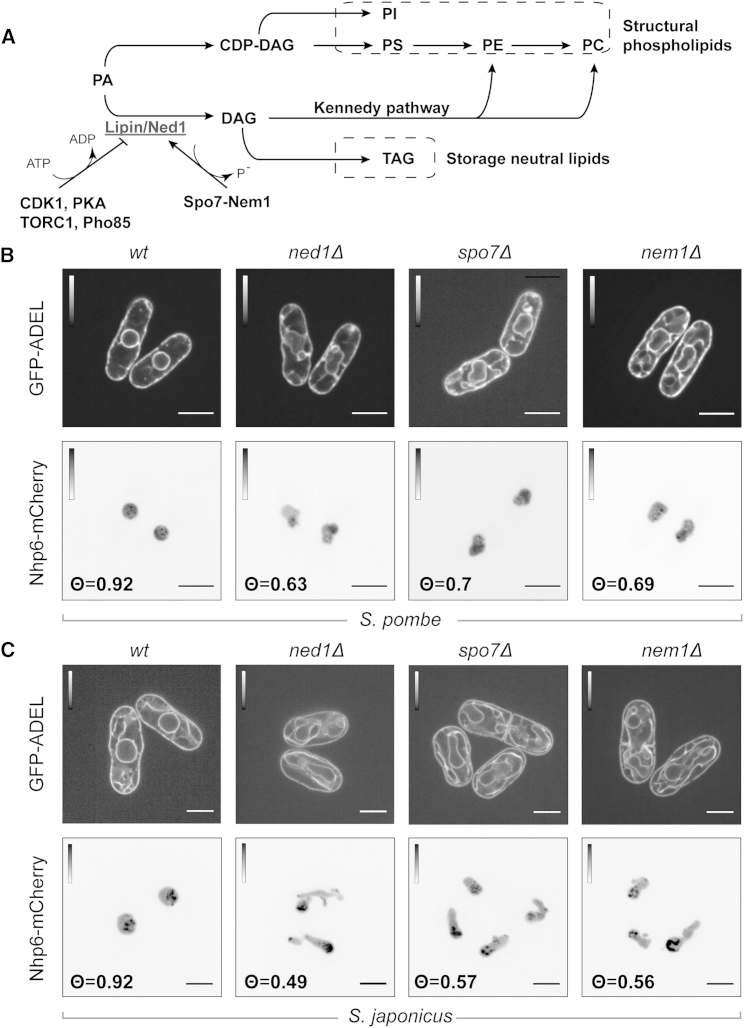
Lipin Dysfunction Leads to NE and ER Expansion Both in *S. pombe* and *S. japonicus* (A) A schematic diagram representing lipid biosynthesis pathways downstream of phosphatidic acid. CDP-DAG, cytidine diphosphate diacylglycerol; DAG, diacylglycerol; PA, phosphatidic acid; PC, phosphatidylcholine; PE, phosphatidylethanolamine; PI, phosphatidylinositol; PS, phosphatidylserine; TAG, triacylglycerol. (B and C) Fluorescence confocal images of *S. pombe* (B) and *S. japonicus* (C) cells of the indicated genotypes co-expressing the ER luminal marker GFP-ADEL and the high mobility group protein Nhp6-mCherry marking nucleoplasm. The Nhp6-mCherry images are inverted. Nuclear circularity indices are presented as Θ values (for both *S. pombe* and *S. japonicus*: WT, n = 51 cells; *ned1Δ*, n = 50 cells; *spo7Δ*, n = 50 cells; and *nem1Δ*, n = 50 cells). The scale bars represent 5 μm. See also Figure S1.

Abnormal NE proliferation in interphase has been linked to changes in phosphatidic acid metabolism [10, 11, 12], suggesting that cell-cycle-dependent mechanisms may similarly regulate NE expansion during mitosis. The phosphatidic acid phosphatase lipin converts phosphatidic acid into diacylglycerol (DAG), which can then be used for production of storage lipids [13] (Figure 1A). When lipin is inactivated in budding yeast, the rate of phospholipid biosynthesis increases and the entire endomembrane system including the NE and the ER expands dramatically [10]. Lipin is regulated negatively by several kinases including Pho85p-Pho80p, Cdc28-CyclinB, PKA, and TORC1 [14, 15, 16, 17, 18, 19, 20] and positively by the ER-localized phosphatase Spo7-Nem1 [12, 17, 21] (Figure 1A).

Lack of the lipin Ned1 or its activator Spo7-Nem1 resulted in steady-state expansion of the entire ER in both *S. pombe* and *S. japonicus*, as visualized by the luminal ER marker GFP-ADEL (Figures 1B and 1C, upper panels; see also [22]). Importantly, the interphase nuclei deviated from their normal spherical shape in the lipin pathway mutants of both species (Figures 1B and 1C, lower panels; see Figure S1A for the nuclear pore marker Nup85-GFP). The nuclei of *S. japonicus* cells lacking Ned1, Spo7, or Nem1 exhibited particularly pronounced flares and low circularity indices (Figures 1C and S1A; see the Supplemental Experimental Procedures for image analysis details). In spite of nuclear membrane expansion in *ned1Δ S. japonicus* cells, the timing of NE breakdown did not change (Figure S1B). Microscopic examination of *ned1Δ S. pombe* and *S. japonicus* cells co-expressing the nucleoplasmic marker Pus1-GFP and the mCherry-tagged nucleolar proteins (Bop1 and Erb1, respectively) showed that, unlike in budding yeast [23], the NE flares in the two fission yeast species were not strictly associated with the nucleolus (Figure S1C). The catalytic mutants of Ned1 (Ned1^D383E/D385E^ in *S. pombe* and Ned1^D422E/D424E^ in *S. japonicus*) exhibited comparable ER expansion and formation of NE flares (Figure S1D), suggesting that the observed endomembrane proliferation was due to a lack of Ned1 enzymatic activity [24]. The growth rates of *ned1Δ* strains decreased in both species as compared to the wild-type controls, with *S. japonicus* being more sensitive to the loss of Ned1 (Figure S1E). Taken together, our results suggest that the general logic of the Ned1-centered circuitry is conserved between the two fission yeast species.

Lipin phosphorylation negatively influences its enzymatic activity [25]. To evaluate Ned1 phosphorylation status in both fission yeasts, we performed immunoprecipitation of the GFP-tagged Ned1 proteins from log-phase cultures of *S. pombe* and *S. japonicus* and analyzed their electrophoretic mobility before and after treatment with protein phosphatase. Consistent with a previous report [22], Ned1-GFP isolated from wild-type *S. pombe* migrated as several bands that collapsed into a faster migrating product upon phosphatase treatment (Figure S2A). Under the same conditions, *S. japonicus* Ned1-GFP showed no detectable electrophoretic mobility shift after treatment with phosphatase (Figure S2A). Yet, Ned1-GFP purified from *spo7Δ* mutants of both *S. pombe* and *S. japonicus* was hyperphosphorylated (Figure S2A), suggesting potential phosphoregulation in both species.

**Figure 2:**
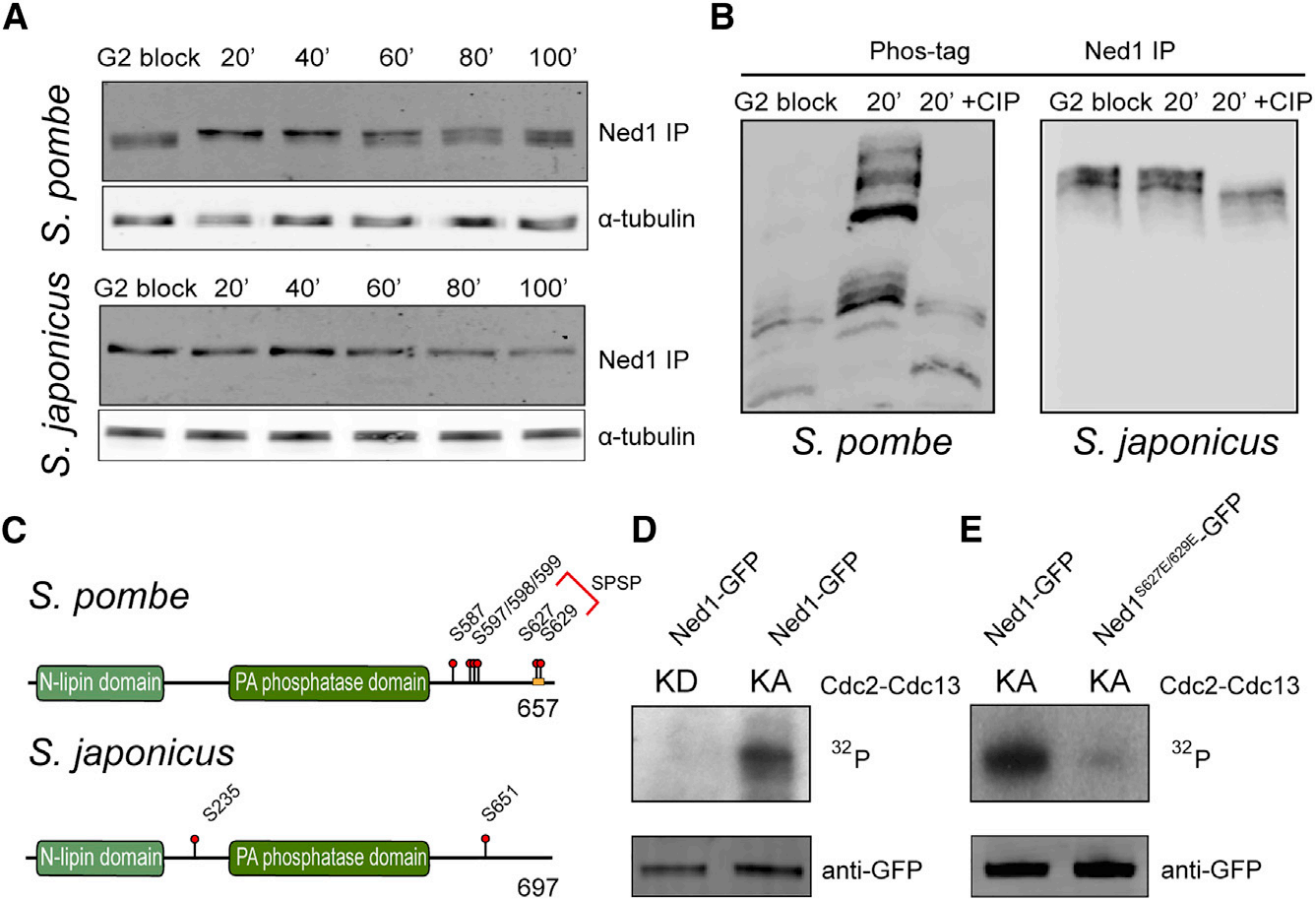
The Lipin Ned1 Is Hyperphosphorylated During Mitosis in *S. pombe*, but Not in *S. japonicus* (A) Cell-cycle synchronization experiments utilizing the temperature-sensitive mutant alleles of *cdc25* (*cdc25-22* for *S. pombe* and *cdc25-D9* for *S. japonicus*). Time after release from the G2/M block is in minutes. Ned1-GFP from each time point was immunoprecipitated and subjected to WB analysis. WB for α-tubulin of input lysates was used as a loading control. (B) Western blot analysis of immunoprecipitated Ned1-GFP from G2/M-blocked and mitotic cells (20 min after release) of *S. pombe* and *S. japonicus*. Samples were separated on 6% SDS-PAGE gel in the presence of Phos-tag. The mitotic samples were also subjected to CIP treatment. (C) Schematic diagrams representing Ned1 S/T phosphorylation sites identified by LC-MS/MS analysis in *S. pombe* and *S. japonicus*. Positions of the evolutionarily conserved N-lipin and catalytic domains are also shown. The red bracket indicates a putative CDK1 phosphorylation site. (D) CDK1 phosphorylates the *S. pombe* lipin Ned1 in vitro. Cdc2-Cdc13 kinase assays were performed using Ned1-GFP purified from G2-arrested *cdc25-22 S. pombe* cells. Ned1-GFP was incubated either with active (KA) or inactive (KD) Cdc2-Cdc13 kinase complexes. Half of the kinase reaction was used to detect phosphorylation by autoradiography (^32^P) and half was used in western blots with anti-GFP antibodies. (E) Serine residues at positions 627 and 629 are essential for phosphorylation of Ned1 by Cdc2-Cdc13 in vitro. The kinase assays were performed using either wild-type or S627E/S629E mutant Ned1 proteins purified from *cdc25-22 S. pombe* cells arrested at G2/M transition. See also Figure S2.

To track changes in Ned1 phosphorylation during the cell cycle, we drove *S. pombe* and *S. japonicus* cells through synchronous mitosis using temperature-sensitive alleles of *cdc25*, a gene controlling the transition from G2 to mitosis [26]. Ned1-GFP was isolated and analyzed by western blotting from cells that were blocked at the G2/M boundary by incubation at the restrictive temperature of 36°C or released into mitosis by decreasing the temperature to 24°C (Figures 2A and S2B). In *S. pombe*, slower migrating forms of Ned1-GFP peaked in mitosis, suggesting that Ned1 was hyperphosphorylated at this stage of the cell cycle (Figure 2A, top panel). Under the same conditions, the electrophoretic migration of *S. japonicus* Ned1-GFP did not change (Figure 2A, bottom panel). To probe mitosis-specific phosphorylation changes further, we performed western blotting of Ned1-GFP isolated from G2-arrested and mitotic extracts in the presence of Phos-tag that allows efficient separation of phosphorylated protein forms [27]. As expected, the *S. pombe* Ned1-GFP exhibited markedly different gel mobility patterns in G2 and mitosis (Figure 2B, left panel). Using this technique, we observed the presence of phosphorylated lipin species in *S. japonicus*. However, the ratio of phosphorylated to non-phosphorylated forms remained comparable between interphase and mitosis (Figure 2B, right panel).

To identify phosphorylation sites on lipin proteins in *S. pombe* and *S. japonicus*, we purified Ned1-GFP from asynchronously growing cultures and performed 2D-liquid chromatography-tandem mass spectrometry (LC-MS/MS). We identified two clusters of phosphorylation in *S. pombe* Ned1 located in its C terminus—S587/S597/T598/S599 and S627/S629. Notably, S627/S629 represents a repeated relaxed S-P consensus motif for phosphorylation by the cyclin-dependent kinase 1 (CDK1). Analysis of Ned1 of *S. japonicus* revealed two possible phosphorylation sites—S235 located between the N-lipin and catalytic domains and S651 at its C terminus (Figures 2C and S2C).

Because *S. pombe* Ned1 is hyperphosphorylated in mitosis, we sought to determine whether CDK1 is directly involved in its phosphoregulation. Ned1-GFP was purified from *S. pombe* cells arrested at the G2/M boundary and subjected to an in vitro CDK1 kinase assay. Autoradiography revealed that ^32^P was readily incorporated into the protein (Figure 2D). Confirming our phosphomapping results, mutating S627/S629 residues to phosphomimetic glutamic acid (Ned1^S627E/S629E^) virtually abolished CDK phosphorylation in vitro (Figure 2E). These data raise the possibility that the lipin proteins are differentially regulated in the two fission yeast species, with the *S. pombe* ortholog undergoing mitotic phosphorylation by CDK1.

**Figure 3:**
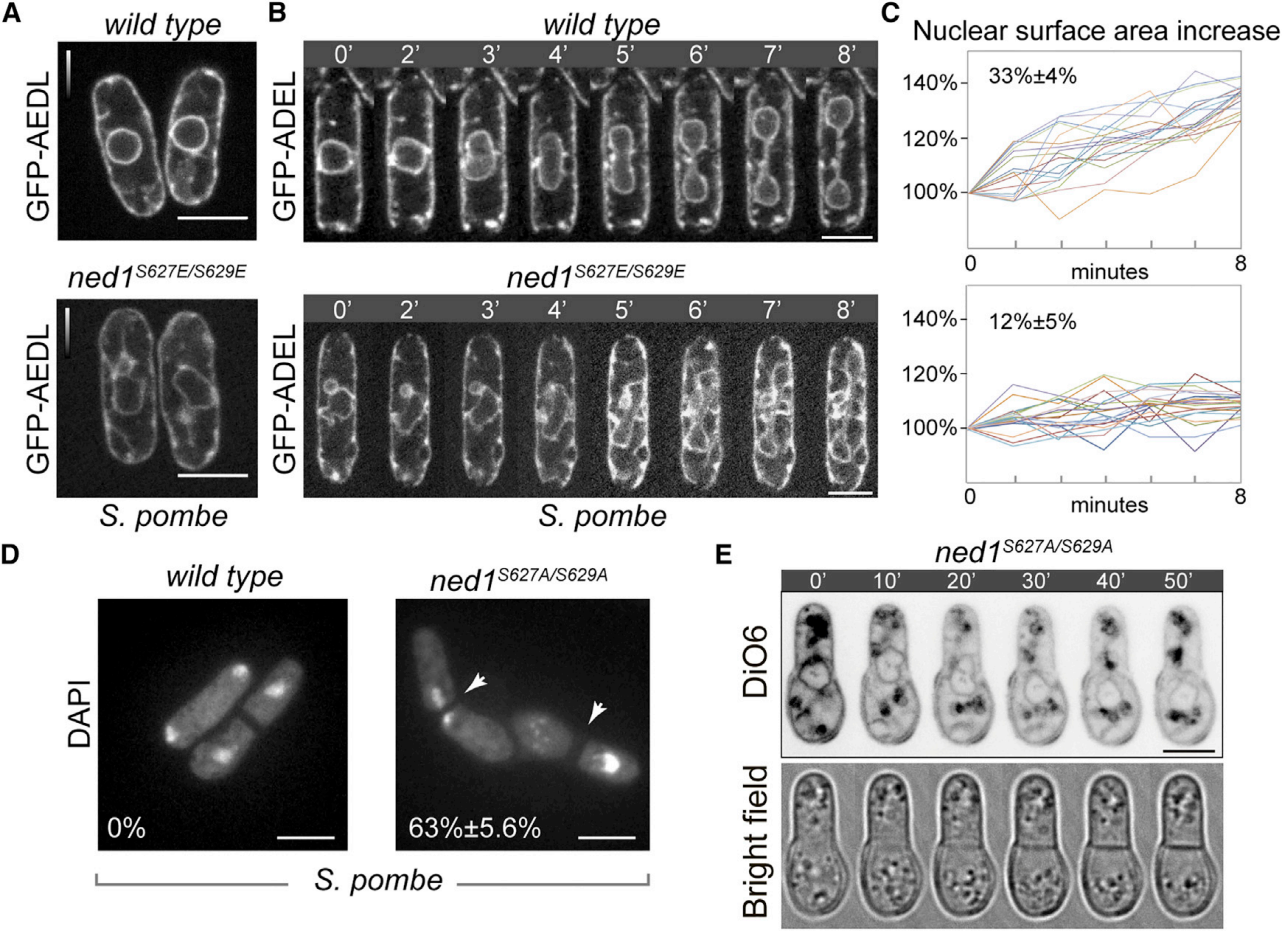
Phosphorylation of the Lipin Ned1 by CDK1 Is Required for Nuclear Division in *S. pombe* (A) Single z-plane confocal images of *S. pombe* expressing the ER marker GFP-ADEL in the wild-type or phosphomimetic Ned1^S627E/S629E^ mutant background. (B) Time-lapse sequences of the wild-type (top) and Ned1^S627E/S629E^ (bottom) cells undergoing mitosis. The ER is marked by GFP-ADEL. Time is in minutes. (C) Plots representing experimentally determined values of the nuclear surface area during nuclear division in the wild-type (top; n = 20) and Ned1^S627E/S629E^ mutant (bottom; n = 20) *S. pombe* cells. (D) Epifluorescence images of fixed and DAPI-stained *S. pombe* cells of the indicated genotypes, where both wild-type and the mutant Ned1 proteins are tagged with GFP. Shown are post-mitotic cells originating from sporulation of the heterozygous diploids carrying either *ned1-gfp* or *ned1*^*S627A/S629A*^-*gfp* integrated at the native *ned1* locus. Arrows indicate cells with “cut” phenotype. The percentage of cells exhibiting a “cut” phenotype is shown (n = 300 cells). (E) Time-lapse sequence of a Ned1^S627A/S629A^ cell undergoing first mitosis after spore germination. Membranes including the NE and the ER are marked by a vital dye DiO6 (top). The phase-contrast image is shown (bottom). Time is in minutes. The scale bars represent 5 μm (A, B, D, and E). See also Figure S3.

To understand the functional consequences of mitotic phosphorylation of Ned1 in *S. pombe*, we mutated the sequence encoding the CDK consensus residues S627/S629 to either alanine or the phosphomimetic glutamic acid at the endogenous locus. *S. pombe* cells solely expressing the phosphomimetic Ned1^S627E/S629E^ variant exhibited expansion of the ER and the misshapen nuclei, indicative of a loss-of-function phenotype (Figure 3A). The nuclear surface area in interphase mutant cells was approximately 34% ± 3% more than the control (n = 10). Because the transient mitotic phosphorylation of Ned1 coincides with an increase in the nuclear surface area in wild-type *S. pombe*, we wondered whether the mutant cells expressing phosphomimetic Ned1 failed to expand the NE further during mitosis. Indeed, unlike in the wild-type cells where the NE surface area grew by 33% ± 4% during mitosis (n = 20 cells; see also [1]), the Ned1^S627E/S629E^ mutant cells showed only a modest 12% ± 5% increase (n = 20; Figures 3B and 3C). We obtained comparable results using *spo7Δ* cells where Ned1 remained hyperphosphorylated (Figure S3A; n = 10). Thus, constitutive phosphorylation of Ned1 on CDK sites in *S. pombe* leads to the steady-state expansion of the NE rather than restricting this process specifically to mitosis.

We repeatedly failed to obtain a haploid strain where Ned1 was refractory to phosphorylation by CDK (Ned1^S627A/S629A^), suggesting that the mutant protein did not support growth. To investigate this possibility, we constructed a diploid strain where one of the wild-type copies of *ned1* was replaced by the *ned1*^*S627A/S629A*^ allele tagged with the selectable auxotrophic marker *ura4+*. The growth rates of diploid *WT/WT* and *WT/ned1*^*S627A/S629A*^ cells at 30°C were comparable although the *WT/ned1*^*S627A/S629A*^ diploids exhibited pronounced lag phase at lower cell densities (Figure S3B). Consistent with the possibility of higher lipin activity leading to accumulation of neutral lipids [28], we observed an increase in lipid droplet abundance in hemizygous diploids (Figure S3C). After induction of sporulation, spores carrying the *ned1*^*S627A/S629A*^ mutant allele were germinated in the absence of uracil. Interestingly, the S627A/S629A mutation caused a highly penetrant mitotic failure manifested as a so-called “cut” phenotype with the division septum bisecting unsegregated chromosomes (Figure 3D; n = 300). As visualized by membrane staining with vital lipophilic fluorescent dye DiOC6 [29], nuclei of S627A/S629A mutant cells initiated anaphase elongation but eventually collapsed and failed to divide (Figure 3E; n = 10). Thus, CDK1-mediated phosphorylation of Ned1 is required for nuclear division in *S. pombe*.

Mutation of two phosphorylation sites identified in *S. japonicus* Ned1 to alanine (Ned1^S235A/S651A^) did not visibly alter NE and ER morphology, with cells undergoing normal mitotic divisions and maintaining viability (Figure S3D). Consistent with higher lipin activity due to its constitutive dephosphorylation, we observed an increase in the number and the size of lipid droplets in Ned1^S235A/S651A^ mutant cells (Figure S3E). On the other hand, *S. japonicus* cells carrying the phosphomimetic Ned1^S235E/S651E^ variant showed NE flares and ER membrane proliferation, somewhat similar to *ned1Δ* mutant (Figure S3D). The mutant cells also exhibited fewer lipid droplets as compared to the control (Figure S3E). These results suggest that, although *S. japonicus* Ned1 functions in lipid droplet biogenesis, its activity is not regulated during mitosis.

**Figure 4:**
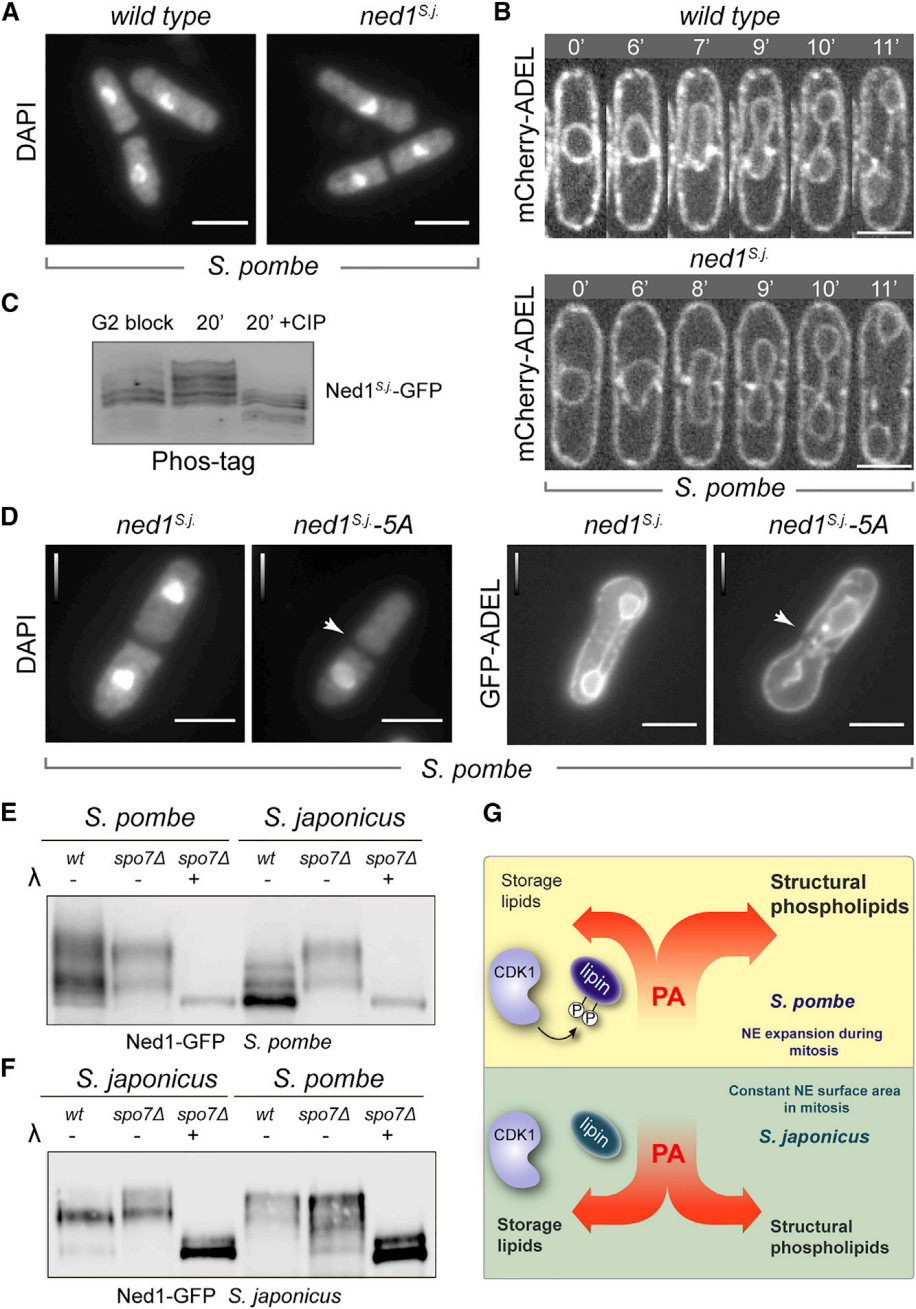
Mitotic Regulation of Ned1 Activity Diverged in the Fission Yeast Clade (A) Single z-plane epifluorescence images of fixed and DAPI-stained *S. pombe* cells expressing either the wild-type Ned1 or its *S. japonicus* ortholog Ned1^*S.j.*^ (n = 300 cells). Shown are post-mitotic cells originating from sporulation of the heterozygous diploids carrying either *ned1-gfp* or *ned*^*S.j.*^*-gfp* integrated at the native *ned1* locus. (B) Time-lapse confocal sequences of *S. pombe* cells expressing either the “wild type” Ned1-GFP (top) or “transplanted” Ned1^*S.j*.^-GFP (bottom) proteins as an only source of Ned1. The ER is labeled by mCherry-ADEL. Time is in minutes. (C) Western blot analysis of immunoprecipitated Ned1^*S.j.*^-GFP purified from either G2-arrested or mitotic *cdc25-22 S. pombe* cells in the presence of Phos-tag. The mitotic sample was also subjected to CIP treatment. (D) (Left panels) Single z-plane epifluorescence images of fixed and DAPI-stained *S. pombe* cells expressing either the wild-type *S. japonicus* ortholog Ned1^*S.j.*^ (left) or the non-phosphorylatable mutant Ned1^*S.j.*^-5A (right) as an only source of Ned1 protein. Shown are post-mitotic cells originating from sporulation of the heterozygous diploids carrying either *ned*^*S.j.*^*-gfp* or *ned*^*S.j.*^*-5A-gfp* integrated at the native *ned1* locus. (Right panels) Single z-plane epifluorescence images of *S. pombe* cells co-expressing the ER marker GFP-ADEL and either Ned1^*S.j*.^-GFP (left) or the non-phosphorylatable Ned1^*S.j.*^-5A-GFP mutant (right) as an only source of Ned1 protein are shown. The experiment was performed as above. (E) Western blot analysis of immunoprecipitated Ned1^*S.p.*^;-GFP purified from *S. pombe* and *S. japonicus* cells of the indicated genotypes in the presence of Phos-tag. The *spo7Δ* samples were also subjected to λ phosphatase treatment. (F) Western blot analysis of immunoprecipitated Ned1^*S.j.*^-GFP purified from *S. japonicus* and *S. pombe* cells of the indicated genotypes in the presence of Phos-tag. The *spo7Δ* samples were also subjected to λ phosphatase treatment. (G) A pictorial model summarizing our current hypothesis on how the NE surface area is controlled during mitosis in the related fission yeast species. The scale bars represent 5 μm (A, B, and D). See also Figure S4.

Interspecies differences in the Ned1 phosphorylation and hence mitotic NE expansion strategies can be due to sequence divergence of the protein itself or its differential regulation by *trans*-acting factors in the two fission yeasts. To distinguish between these possibilities, we interchanged the *ned1* open reading frames (ORFs) of *S. pombe* and *S. japonicus*, leaving the host-specific untranslated gene regions intact. Interestingly, germination of *S. pombe* spores carrying the *ned1*^*S. japonicus*^::*ura4+* (*ned1*^*S.j.*^) allele as its sole *ned1* copy yielded healthy cells exhibiting normal chromosome partitioning as judged by staining the DNA with DAPI (Figure 4A; n = 300). Live cell imaging of mCherry-ADEL-expressing *S. pombe* cells carrying the transplanted Ned1^*S.j.*^ confirmed that mitosis was phenotypically normal (Figure 4B). Similarly, in *S. japonicus*, swapping the native Ned1 with its *S. pombe* ortholog Ned1^*S. pombe*^ (*ned1*^*S.p.*^) did not lead to obvious differences in mitotic nuclear dynamics (Figure S4A).

Indeed, the population-doubling times were comparable between the lipin-swapped and the wild-type cultures (2.34 ± 0.18 versus 2.31 ± 0.11 hr for *S. pombe* wild-type and Ned1^*S.j.*^ and 1.8 ± 0.22 versus 1.84 ± 0.13 hr for *S. japonicus* wild-type and Ned1^*S.p.*^). The fact that the two lipins could substitute for each other when expressed in a heterologous system indicates that the regulatory modes rather than the lipin protein properties diverged between the two fission yeasts.

The gel migration patterns of Ned1^*S.j.*^-GFP were markedly different in G2-arrested and mitotic *S. pombe* cells, indicating that the transplanted Ned1 protein could be subject to *S. pombe*-specific mitotic phosphorylation events (Figures 4C, S4B, and S4C). Recombinant Ned1^*S. japonicus*^ could be phosphorylated by Cdk1 in vitro (Figure S4D), and Ned1^*S. japonicus*^ has several putative CDK phosphorylation sites, including five at its C terminus, S499, S538, T620, T638, and S651, suggesting that similar phosphoregulation could occur in *S. pombe* cells. Replacement of these serine and threonine residues with non-phosphorylatable alanines rendered the transplanted Ned1^*S.j.*^ defective in supporting mitotic division in *S. pombe* cells. Similar to the phenotype observed in *S. pombe ned1*^*S627A/S629A*^ mutant (Figure 3D), many germinated spores carrying *ned1*^*S.j.*^*-5A*::*ura4+* allele failed to properly partition chromosomes and divide the nucleus in the first mitotic division (Figure 4D; 43% ± 7% cells exhibited “cut” phenotype; n = 300). On the other hand, the phosphomimetic variant Ned1^*S.j.*^-5E caused a loss-of-function phenotype in *S. pombe*, with mutant cells exhibiting constitutive ER and nuclear membrane expansion (Figure S4E). When mutated in *S. japonicus*, the same phosphomimetic mutant triggered ER-NE expansion during interphase and decreased lipid droplet formation (Figures S4F and S4G). Introduction of the 5A mutant in *S. japonicus* did not cause mitotic abnormalities but produced cells with more lipid droplets (Figures S4F and S4G), reminiscent of the phenotype observed in *ned1*^*S235A/S651A*^ genetic background (Figure S3E).

To address the potential contribution of Spo7-Nem1 phosphatase to lipin regulation in the two species, we analyzed the electrophoretic migration of Ned1^*S. pombe*^ in the presence of Phos-tag in *S. pombe* and *S. japonicus* asynchronous wild-type and *spo7*Δ cultures. When expressed in its native wild-type environment, Ned1^*S. pombe*^ migrated as multiple phospho-forms, suggesting potential phosphoregulation by multiple inputs (Figure 4E). Strikingly, when transplanted in *S. japonicus* cells, Ned1^*S. pombe*^ acquired a distinct electrophoretic mobility pattern, consistent with the pronounced presence of a dephosphorylated form. Introducing the transplanted protein into the *spo7*Δ background led to increased phosphorylation (Figure 4E). This suggests that the *S. pombe* lipin, normally a highly phosphorylated protein, is subject to massive dephosphorylation by Spo7-Nem1 phosphatase in *S. japonicus*.

In a reciprocal experiment, we analyzed the phospho-state of the *S. japonicus* lipin in both yeasts. We consistently detected a major phospho-form together with a dephosphorylated species of lipin in *S. japonicus* wild-type cells. As expected, the lack of Spo7 led to hyperphosphorylation of Ned1 (Figure 4F). When transplanted into *S. pombe*, Ned1^*S. japonicus*^ migrated differently than it did in *S. japonicus,* consistent with differential phosphoregulation in this species (Figure 4F).

Taken together, our results suggest that the fundamental differences between mitotic strategies of the two fission yeasts might depend on divergence of the regulatory networks controlling lipin activity. Phosphorylation of Ned1 by CDK1 in *S. pombe* allows this organism to inactivate the bulk of the enzyme specifically in mitosis. Lipin inactivation presumably triggers increased phospholipid production required for massive NE expansion during “closed” nuclear division. On the other hand, overall phosphorylation status of lipin in *S. japonicus* remains constant throughout the cell cycle (Figure 4G). This does not mean that lipin activity is not regulated by phosphorylation in this organism (see Figures 2B, 2C, S3D, and S3E)—only that such phosphoregulation is not entrained to the mitotic cell cycle. Indeed, lipin activity must be dynamically controlled within a cell to produce DAG in a spatially restricted manner, supporting lipid droplet biogenesis [30], vacuole homeostasis [31], and other biosynthetic and signaling events [25]. At steady state, the overall lipin phosphorylation and, hence, its activation status may reflect the opposing kinase and phosphatase activities. Mitotic transition to the predominantly phosphorylated form of lipin in *S. pombe* could be due to a disruption in this balance, for instance because of concurrent inactivation of the Spo7-Nem1 phosphatase. The fact that the transplanted *S. pombe* lipin is more dephosphorylated in *S. japonicus* suggests a powerful contribution of phosphatase in this species (Figure 4E). Alternatively, it is also possible that pre-existing protein phosphorylation could prevent CDK-dependent modifications of lipin [32].

As compared to *S. pombe*, lipin deficiency in *S. japonicus* produces considerably stronger expansion of the NE-ER system and has a more deleterious impact on cellular growth rate (Figures 1 and S1). Different outputs downstream of lipin inactivation may necessitate keeping the bulk of lipin relatively active at all times in cycling *S. japonicus* cells. Work in budding yeast uncovered crosstalk between lipin function and transcriptional control of a cohort of inositol-responsive lipid biosynthesis genes [25], but this gene regulation circuitry is not conserved between budding and fission yeasts. Mammalian lipins have been also implicated in transcriptional regulation of genes involved in fatty acid oxidation and adipocyte differentiation [33]. Determining relative contributions of the lipin enzymatic and gene regulation modalities in both *S. pombe* and *S. japonicus* and deducing species-specific response patterns to genetic perturbations of the lipin-centered circuitry will be important to illuminate possible physiological and metabolic foundations for the distinct lipin network topologies within the clade.

Metazoan lipins and the Spo7-Nem1-related phosphatases are important for NE/ER biogenesis and mitotic remodeling [21, 34, 35, 36, 37] and function at the intersection of several metabolic pathways [33, 38]. The two fission yeast species with their naturally divergent NE expansion strategies provide an excellent system yielding fundamental insights into lipin function and control of phosphatidic acid flux potentially relevant to all eukaryotes.

## Experimental Procedures

### Yeast Strains and Culture Conditions

*S. pombe* and *S. japonicus* strains used in this study and their genotypes are listed in Table S1. Genetic methods, culturing conditions, and transformation procedures for both species have been described previously [39, 40, 41]. For details of strain construction, protein biochemistry, and imaging methods, please see the Supplemental Experimental Procedures.

## Author Contributions

M.M. did in vivo experiments and co-wrote the manuscript. Y.G. generated a number of *S. japonicus* strains including several fluorescent tags, a conditional *cdc25* mutant, and a *ned1* replacement strain. J.-S.C. performed in vitro kinase assays and mass spectrometry. J.R.B. analyzed mass spectrometry data and edited the manuscript. K.L.G. provided input into the design and interpretation of experiments and edited the manuscript. S.O. guided the project and co-wrote the manuscript.

## Supplemental Information

Document S1. Supplemental Experimental Procedures, Figures S1–S4, and Table S1

Document S2. Article plus Supplemental Information
