# Supplementary material for: Temporal Regulation of Lipin Activity Diverged to Account for Differences in Mitotic Programs
Source: Curr Biol. 2016 Jan 25;26(2):237–43. doi: 10.1016/j.cub.2015.11.061 (PMC4728079; doi:10.1016/j.cub.2015.11.061)
Supplement: Document S1. Supplemental Experimental Procedures, Figures S1–S4, and Table S1 [file mmc1.pdf]

Current Biology

Supplemental Information

Temporal Regulation of Lipin Activity Diverged to Account for Differences in Mitotic Programs

Maria Makarova, Ying Gu, Jun-Song Chen, Janel Renée Beckley, Kathleen Louise Gould, and Snezhana Oliferenko

Supplemental Figures

Makarova *et al.*, Fig. S1

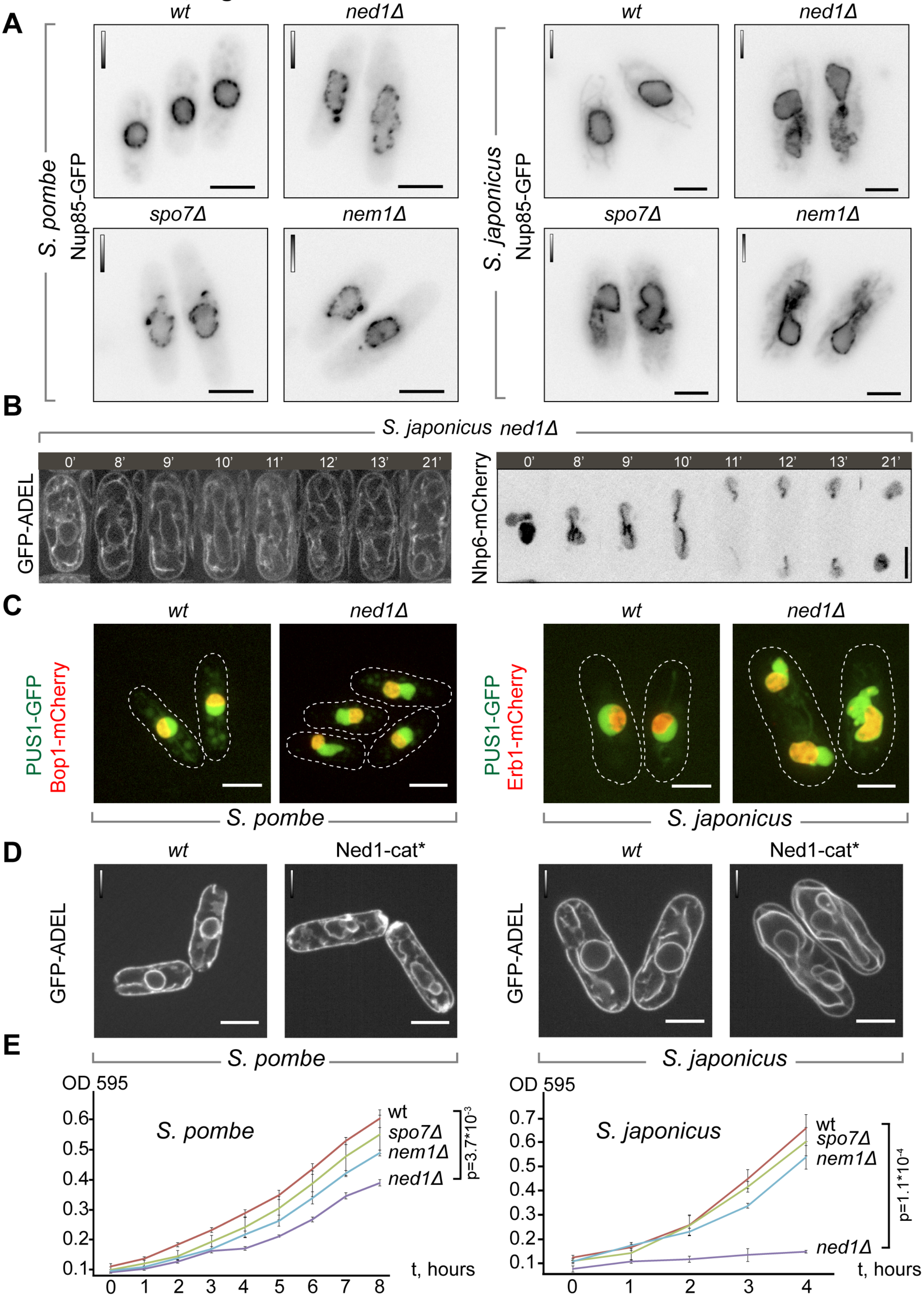

Makarova *et al.*, Fig. S2

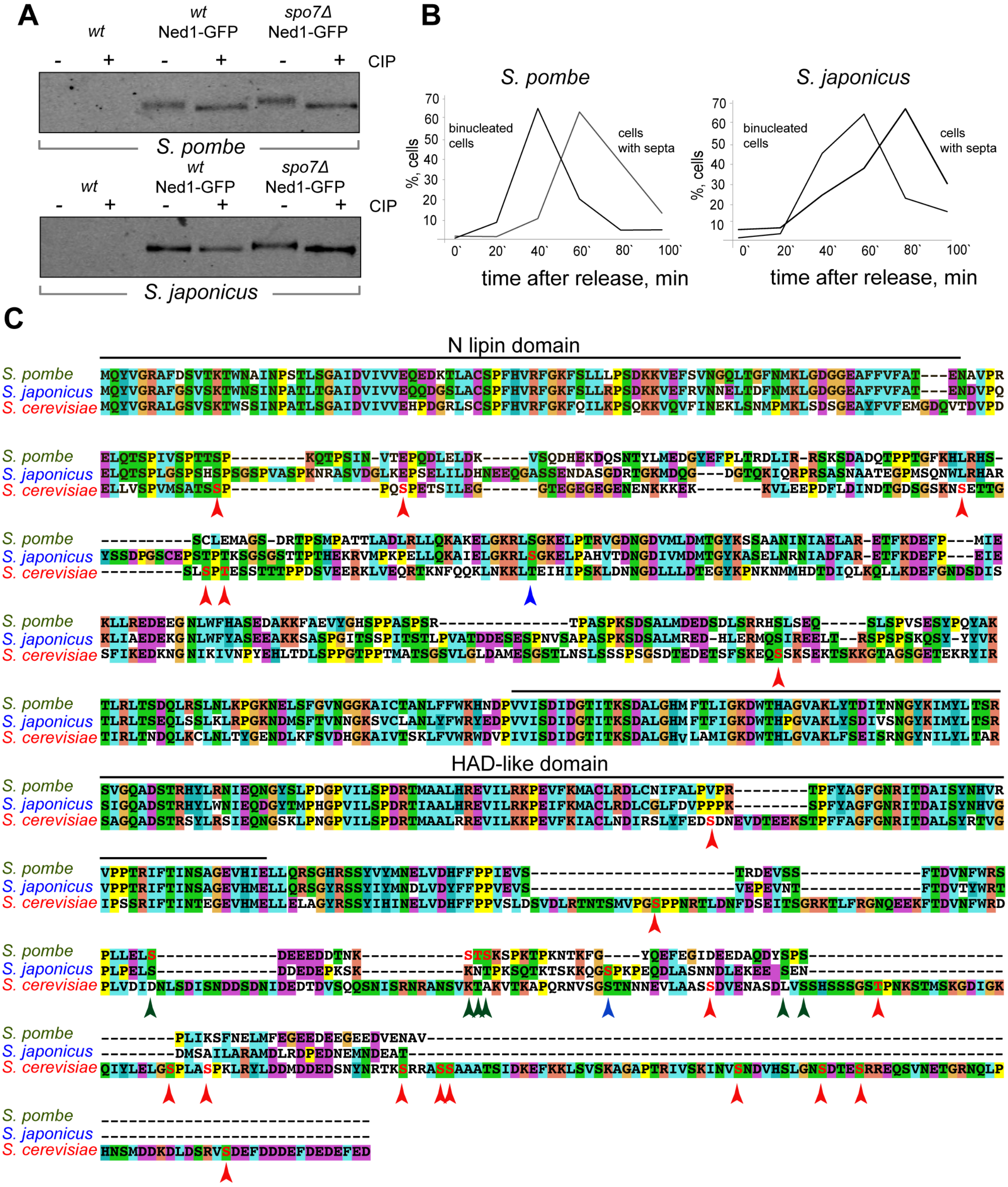

Makarova *et al.*, Fig. S3

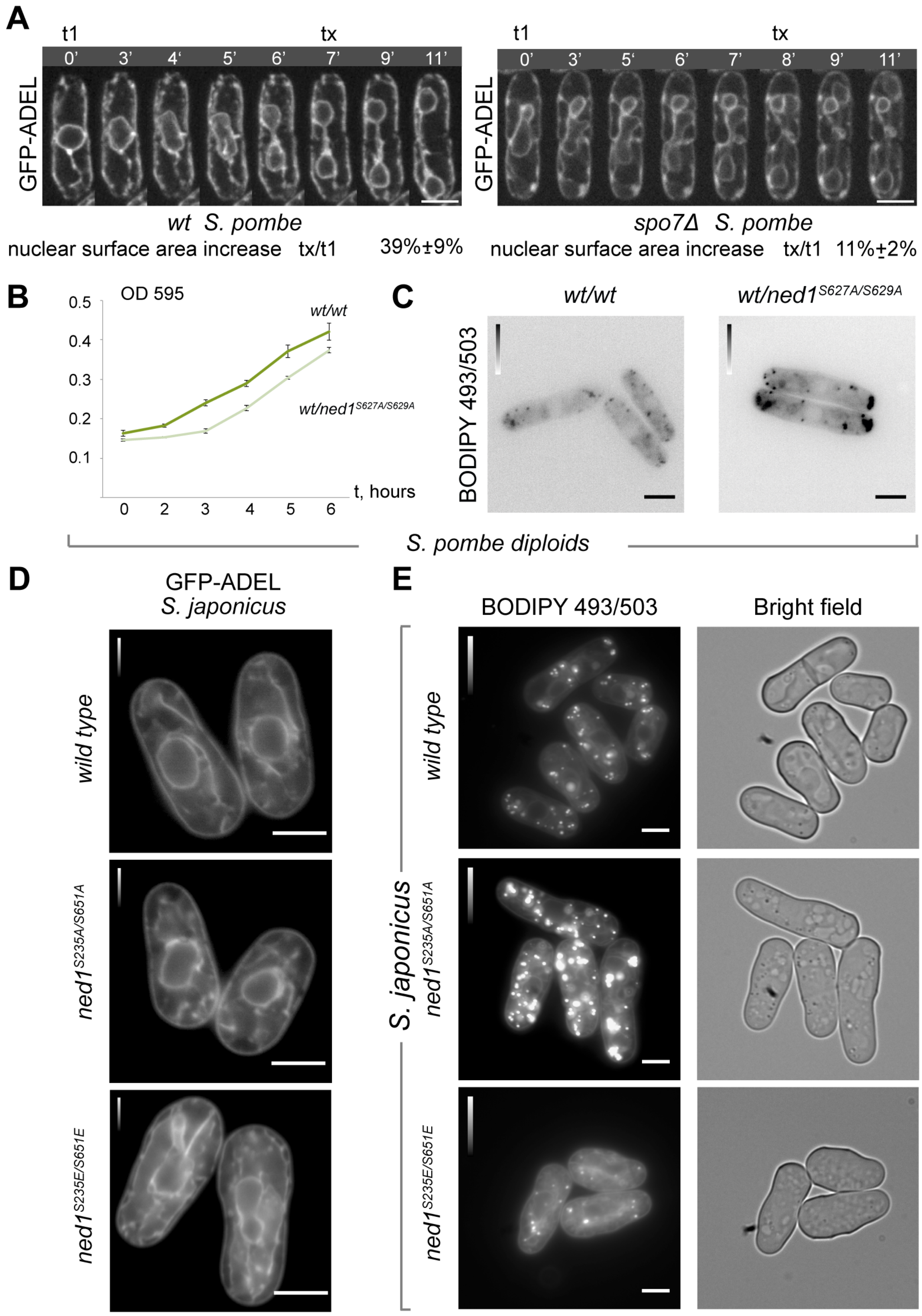

Makarova *et al.*, Fig. S4

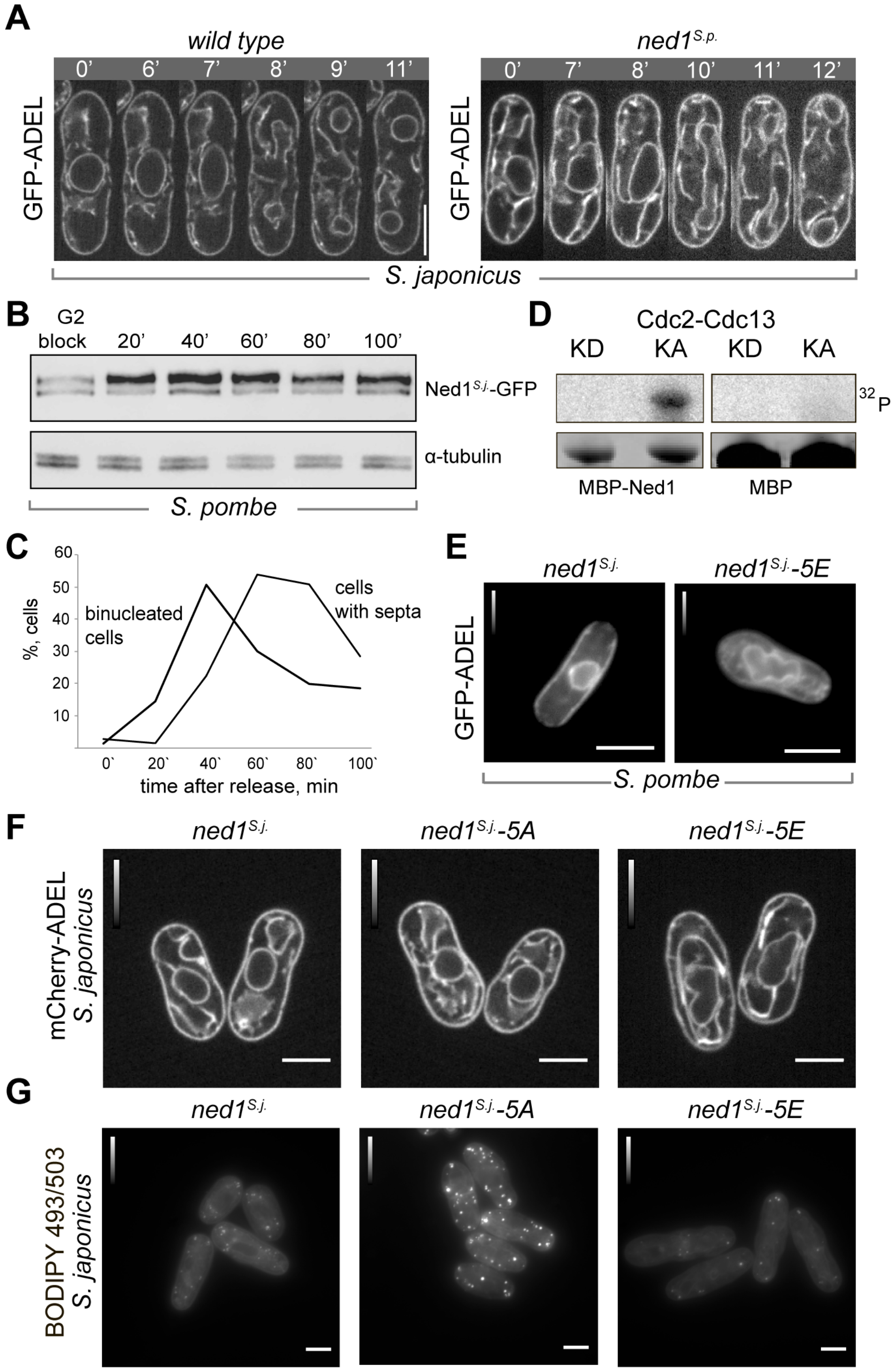

Supplemental Legends

Figure S1, related to Figure 1. (**A**) Inverted single z-plane epifluorescence images of *S. pombe* and *S. japonicus* cells of indicated genotypes expressing the nuclear pore marker Nup85-GFP. (**B**) Time-lapse sequence of GFP-ADEL (left) and Nhp6-mCherry (right) labeled *ned1Δ S. japonicus* cell undergoing mitosis. Note that the NE breaks down in anaphase similar to the control (compare with Fig. S4A). Time is in minutes. (**C**) Maximum projections of confocal z-stack images of *S. pombe* and *S. japonicus* cells of indicated genotypes co-expressing the nucleoplasmic marker Pus1-GFP and the mCherry-tagged nucleolar protein (Bop1 for *S. pombe* and Erb1 for *S. japonicus*). Note that NE extensions are not strictly associated with the nucleolus. **(D)** Single z-plane confocal images of *S. pombe* and *S. japonicus* cells of indicated genotypes expressing the luminal ER marker GFP-ADEL. $Ned1^{cat*}$ are catalytic mutants in both species. (**A**-**D**) Scale bars, 5μm. (**E**) Growth curves of *S. pombe* and *S. japonicus* cultures of indicated genotypes. Cells were grown at 30°C in the rich YES medium, with OD_{595} measurements performed each hour. Error bars represent standard deviation between three independent experiments. Unpaired t-test was used to obtain the p-values.

Figure S2, related to Figure 2. (A) Western blot (WB) analysis of immunoprecipitated Ned1-GFP from strains of indicated genotypes. CIP, treatment with alkaline calf intestinal phosphatase. (**B**) Exponentially growing *cdc25-22 S. pombe* and *cdc25-D9 S. japonicus* cells were blocked at the G2/M transition by incubating at 36°C for 3.5 hours. Cells were released into mitosis by cooling cultures to 24°C. Samples were taken at consecutive time points as indicated and fixed with paraformaldehyde. Septation index was counted in bright-field images; DAPI was used to evaluate the number of bi-nucleate anaphase cells (for each time point 400 cells were calculated). (**C**) Sequence alignment of the lipin proteins from *S. pombe*, *S. japonicus* and *S. cerevisiae*. Sequences were aligned using Clustal X ver. 2.1 [S1]. Assigned colors of the specific residues are based on alignment consensus. Arrows (*S. pombe*, green; *S. japonicus*, blue; *S. cerevisiae*, red) indicate phosphorylation sites identified by LC-MS/MS analyses. N-lipin and catalytic domains are indicated.

Figure S3, related to Figure 3. (A) Time-lapse sequences of the wild type (*left,* n=10) and *spo7Δ* (*right,* n=10) *S. pombe* cells undergoing mitosis. The ER is labeled with GFP-ADEL. Time is in minutes. Numbers indicate average experimentally determined values of the nuclear surface area increase between indicated time points. (**B**) Growth curves of diploid *wt/wt* and *wt/ned1*^{S627AS629A} *S. pombe* cultures. Cells were grown at 30°C in the synthetic EMM medium, with OD_{595} measurements performed each hour. Error bars represent standard deviation between three independent experiments. (**C**) Maximum projection epifluorescence images of diploid *wt/wt* and *wt/ned1*^{S627AS629A} *S. pombe* cells stained with the neutral lipid dye BODIPY 493/503. Shown are inverted images. (**D**) Single z-plane epifluorescence images of wild type, Ned1-S235A/S651A-GFP and Ned1-S235E/S651E-GFP *S. japonicus* cells expressing the ER marker GFP-ADEL. (**E**) Maximum projection epifluorescence images of wild type, Ned1-S235A/S651A-GFP and Ned1-S235E/S651E-GFP *S. japonicus* cells stained with the neutral lipid dye BODIPY 493/503. (**A**, **C**, **D**, **E**) Scale bars, 5 µm.

Figure S4, related to Figure 4. (**A**) Time-lapse sequences of mitotic *S. japonicus* cells co-expressing the ER marker GFP-ADEL together with the "wild type" Ned1-GFP (*top,* n=5) or its "transplanted" *S. pombe* ortholog (*Ned1$^{S.p.-}$GFP*) (*bottom,* n=4). (**B**) G2 block and cell cycle release of *cdc25-22* mutant *S. pombe* cells expressing Ned1$^{S.j.}$-GFP as an only source of Ned1. At each time point, Ned1$^{S.j.}$-GFP was immunoprecipitated and subjected to WB analysis. WB for α-tubulin present in input lysates was used as a loading control. (**C**) As a control for an experiment shown in (**B**), we provide quantification of the cell cycle block and release experiment. Exponentially growing *cdc25-22 S. pombe* cells expressing Ned1$^{S.j.-}$GFP were blocked at the G2/M transition by incubating at 36°C for 3.5 hours. Cells were released into mitosis by cooling cultures to 24°C. Samples were taken at consecutive time points as indicated and fixed with paraformaldehyde. Septation index was counted in brightfield images; DAPI was used to evaluate the number of bi-nucleate anaphase cells (for each time point 400 cells were calculated). (**D**) CDK1 phosphorylates the *S. japonicus* lipin Ned1 *in vitro*. Cdc2-Cdc13 kinase assays were performed using MBP-Ned1 purified from *E.coli*. MBP-Ned1 was incubated either with active (KA) or inactive (KD) Cdc2-Cdc13 kinase complexes. Kinase assays with MBP alone are shown as a control. (**E**) Single z-plane epifluorescence images of *S. pombe* cells co-expressing the ER marker GFP-ADEL and either the "wild type" or phosphomimetic mutant of Ned1$^{S.j.}$-5E-GFP as an only source of Ned1 protein. (**F**) Single z-plane confocal images of *S. japonicus* cells of indicated genotypes expressing ER marker mCherry-ADEL. (**G**) Maximum projection epifluorescence images of *S.*

japonicus cells of indicated genotypes stained with the neutral lipid dye BODIPY 493/503. (**A**, **E**, **F**, **G**) Scale bars, 5 μm.

Supplemental Table S1

S. pombe strains

Figure	Genotype	Collection No.
1B	*bip1-GFP-ADEL::ura4+ Nhp6-mCherry::ura4+ ade6-? leu1-32 ura4-D18*	SO7587
1B	*spo7Δ::ura4+ bip1-GFP-ADEL::ura4+ Nhp6-mCherry:ura4+ ade6-? leu1-32 ura4-D18*	SO7588
1B	*ned1 Δ:ura4+ bip1-GFP-ADEL::leu1+ Nhp6-mCherry:ura4+ ade6-? leu1-32 ura4-D18*	SO8006
1B	*nem1 Δ:ura4+ bip1-GFP-ADEL::leu1+ Nhp6-mCherry:ura4+ ade6-? leu1-32 ura4-D18*	SO7943
S1A	*spo7Δ::ura4+ nup85-GFP::ura4+ ade6-? leu1-32 ura4-D18*	SO7475
S1A	*nem1Δ:ura4+ nup85-GFP::ura4+ ade6-? leu1-32 ura4-D18*	SO7995
S1A	*ned1Δ:ura4+ nup85-GFP::ura4+ ade6-? leu1-32 ura4-D18*	SO7476
S1A	*nup85-GFP::ura4+ ade6-210 leu1-32 ura4-D18*	SO3985
S1C	*ned1Δ:ura4+ Pus1-GFP:ura4+ Bop1-mCherry:ura4+ ade6-? leu1-32 ura4-D18*	SO8105
S1C	*Pus1-GFP:ura4+ Bop1-mCherry:ura4+ ade6-? leu1-32 ura4-D18*	SO8100
S1D	*bip1-GFP-ADEL::leu1+ Ned1*$^{D382E/D385E}$*-GFP::ura4+ ade6-? leu1-32 ura4-D18*	SO7746
S1D, S3A	*bip1-GFP-ADEL::leu1+ ade6-? leu1-32 ura4-D18 h+*	SO4808
S1E	*ade6-210 leu1-32 ura4-D18 h+*	SO2865
S1E	*spo7Δ::ura4+ ade6-? leu1-32 ura4-D18 h+*	SO7472
S1E	*nem1Δ::ura4+ ade6-? leu1-32 ura4-D18 h+*	SO8002
S1E	*ned1Δ::ura4+ ade6-? leu1-32 ura4-D18 h+*	SO7909
S2A, 4E	*Ned1-GFP::ura4+ spo7Δ::ura4+ ade6-? leu1-32 ura4-D18*	SO7634
S2A, 2C, 2E, 4E	*Ned1-GFP::ura4+ ade6-? leu1-32 ura4-D18*	SO7644
2A, 2B, 2D, S2B	*Ned1-GFP::ura4+cdc25-22:ura4+ ade6-? leu1-32 ura4-D18*	SO7645
2E	*Ned1*$^{S627E/S629E}$*-GFP::ura4+ ade6-? leu1-32 ura4-D18*	SO7921
3A, 3B, 3C, S4D	*Ned1-GFP:ura4+ bip1-GFP-ADEL::leu1+ ade6-? leu1-32 ura4-D18*	SO7796
3A, 3B, 3C	*Ned1*$^{S627E/S629E}$*-GFP::ura4+ bip1-GFP-ADEL::leu1+ ade6-? leu1-32 ura4-D18*	SO7922
3D, S3B, 4A	Heterozygous diploid *Ned1-GFP::ura4+ ade6-210 ade216 leu1-32 ura4-D18*	SO7653
3D, 3E, S3B	Heterozygous diploid *Ned1*$^{S627A/S629A}$*-GFP::ura4+ ade6-210 ade216 leu1-32 ura4-D18*	SO7905
S3A	*spo7Δ::ura4+ bip1-GFP-ADEL::leu1+ ade6-? leu1-32 ura4-D18*	SO7643
4A, 4F	*Ned1-GFP* $^{S.japonicus}$*::ura4 ade6-? leu1-32 ura4-D18*	SO7747
4B	*bip1-mCherry-ADEL::leu1+ ade6-? leu1-32 ura4-D18*	SO5609
4B	*Ned1-GFP* $^{S.japonicus}$*::ura4+ bip1-mCherry-ADEL::leu1+ ade6-? leu1-32 ura4-D18*	SO7793
4D	Heterozygous diploid *Ned1*$^{S.japonicus}$*-GFP::ura4+ bip1-GFP-ADEL::leu1+ ade6-210 ade216 leu1-32 ura4-D18*	SO7985
4D	Heterozygous diploid *Ned1*$^{S.japonicus}$*-5A-GFP::ura4+ bip1-GFP-ADEL::leu1+ ade6-210 ade216 leu1-32 ura4-D18*	SO7986
S4D	*Ned1*$^{S.japonicus}$*-5E-GFP::ura4+ bip1-GFP-ADEL::leu1+ ade6-210 ade216 leu1-32 ura4-D18*	SO7989

4C, S4B, S4C	Ned1$^{S.japonicus}$-GFP::ura4+ cdc25-22::ura4+ ade6-210 ade216 leu1-32 ura4-D18	SO7791
4F	Ned1-GFP $^{S.japonicus}$::ura4 ade6-? spo7Δ::ura4+ leu1-32 ura4-D18	SO7898

S. japonicus strains

**Figure**	**Genotype**	**Collection No.**
1C	bip1-GFP-ADEL::ura4+ Nhp6-mCherry::ura4+ ura4sj-D3 ade6sj-domE	SOJ501
1C	spo7Δ::ura4+ bip1-GFP-ADEL::ura4+ Nhp6-mCherry::ura4+ura4sj-D3 ade6sj-domE	SOJ1506
1C, S1B	ned1Δ:: kan^{R} bip1-GFP-ADEL::ura4+ Nhp6-mCherry::ura4+ ura4sj-D3 ade6sj-domE	SOJ2197
1C	nem1Δ::ura4+ bip1-GFP-ADEL::ura4+ Nhp6-mCherry::ura4+ura4sj-D3 ade6sj-domE	SOJ2194
S1A	Nup85-GFP::ura4+ura4sj-D3 ade6sj-domE	SOJ54
S1A	nem1Δ::ura4+ nup85-GFP::ura4+ ura4sj-D3 ade6sj-domE	SOJ2193
S1A	spo7Δ::ura4+ nup85-GFP::ura4+ ura4sj-D3 ade6sj-domE	SOJ1504
S1A	ned1Δ::kan^{R} nup85-GFP::ura4+ ura4sj-D3 ade6sj-domE	SOJ1820
S1C	Pus1-GFP:ura4+ Erb1-mCherry::ura4+ ura4sj-D3 ade6sj-domE	SOJ2408
S1C	ned1Δ::kan^{R} Pus1-GFP:ura4+ Erb1-mCherry::ura4+ ura4sj-D3 ade6sj-domE	SOJ2483
S1D	Ned1$^{D422E/D424E-}$GFP::ura4 bip1-GFP-ADEL::ura4+ ura4sj-D3 ade6sj-domE	SOJ2012
S1D	bip1-GFP-ADEL::ura4+ ura4sj-D3 ade6sj-domE	SOJ495
S1E	ade6sj-domE ura4sj-D3 h+	SOJ88
S1E	spo7Δ::ura4+ h+ ade6sj-domE ura4sj-D3	SOJ1502
S1E	ned1Δ::kan^{R} h+ ade6sj-domE ura4sj-D3	SOJ1778
S1E	nem1Δ::ura4+ h+ ade6sj-domE ura4sj-D3	SOJ2191
2C, 4F, S2A, S3E, S4G	Ned1-GFP::ura4+ h+ ade6sj-domE ura4sj-D3	SOJ1410
4F, S2A	Ned1-GFP::ura4+ spo7Δ::ura4+ ura4sj-D3 ade6sj-domE	SOJ1879
2A, 2B, S2B	Ned1-GFP::ura4+ cdc25-D9::ura4+ ura4sj-D3 ade6sj-domE	SOJ1500
S3D,	Ned1$^{S235A/S651A}$-GFP::ura4+ bip1-GFP-ADEL::ura4+ ura4sj-D3 ade6sj-domE	SOJ2131
S3D,	Ned1$^{S235E/S651E}$-GFP::ura4+ bip1-GFP-ADEL::ura4+ ura4sj-D3 ade6sj-domE	SOJ2139
S3D, S4A, S4F	Ned1-GFP::ura4+ bip1-GFP-ADEL::ura4+ ura4sj-D3 ade6sj-domE	SOJ1453
S3E	Ned1$^{S235A/S651A}$-GFP::ura4+ ura4sj-D3 ade6sj-domE	SOJ2130
S3E	Ned1$^{S235E/S651E}$-GFP::ura4+ ura4sj-D3 ade6sj-domE	SOJ2137
S4E	Ned1$^{S. pombe}$-GFP::ura4+ ura4sj-D3 ade6sj-domE	SOJ2482
S4A	Ned1$^{S. pombe}$-GFP::ura4+ bip1-GFP-ADEL::ura4+ ura4sj-D3 ade6sj-domE	SOJ2486
S4F	Ned1^{-5A}-GFP::ura4+ bip1-mCherry-ADEL::ura4+ ura4sj-D3 ade6sj-domE	SO1793
S4F	Ned1^{-5E}-GFP::ura4+ bip1-mCherry-ADEL::ura4+ ura4sj-D3 ade6sj-domE	SO1799
S4G	Ned1-^{5A}-GFP::ura4+ ura4sj-D3 ade6sj-domE	SO1788
S4G	Ned1-^{5E}-GFP::ura4+ ura4sj-D3 ade6sj-domE	SO1790
S4E	Ned1$^{S. pombe}$-GFP::ura4+ spo7Δ::ura4+ ura4sj-D3 ade6sj-domE	SOJ2485

Supplemental Experimental Procedures

Strains and reagents

All strains used in this study are listed in Supplemental Table S1. *spo7*Δ and *nem1*Δ strains of both *S. japonicus* and *S. pombe* and the *ned1*Δ *S. pombe* mutant were constructed using the pJK210 plasmid backbone carrying the species-specific *ura4* cassettes flanked by 5' and 3' UTRs of respective genes. The *ned1*Δ *S. japonicus* strain was constructed using a standard PCR-based recombination method using KanMX6 as a selection marker. Ned1$^{D383E/D385E}$, Ned1$^{S627A/S629E}$, Ned1$^{S627E/S629E}$ mutants for *S. pombe* and Ned1$^{D422E/D424E}$, Ned1$^{S235A/S651A}$, Ned1$^{S235E/651E}$ for *S. japonicus* were generated by single end recombination using pJK210 backbone plasmids carrying the species-specific *ura4* cassettes. Replacement of *ned1* ORF in *S. pombe* with its *S. japonicus* ortholog was performed using a construct containing the 5'UTR and 3'UTR of ned1$^{S.pombe}$ that flanked the *ned1* ORF$^{S.japonicus}$. Replacement of *ned1* ORF$^{S.pombe}$ with mutant versions of *ned1*$^{S.japonicus}$ was performed similarly, with S499, S538, T620, T638 and S651 mutated to either alanine or glutamic acid. Replacement of *ned1* ORF in *S. japonicus* with its *S. pombe* ortholog was performed using a construct containing the 5'UTR and 3'UTR of ned1$^{S.japonicus}$ that flanked the *ned1* ORF$^{S.pombe}$. All Ned1 variants were tagged with GFP at C-terminus.

Please note that our data amend the automatic intron-exon annotation for the *S. japonicus ned1* gene provided by the Broad Institute [S2]. The second annotated 150bp-long intron is retained, resulting in insertion of additional 50 amino acids in a region between the N-lipin and HAD-like domains. D*e novo*

transcriptome assembly of RNA-seq data from both species confirmed that the second intron is retained in *S. japonicus* but not in *S. pombe* (data not shown). We did not observe any evidence for alternative splicing in the mitotic cycle. Sequencing of *ned1* cDNA isolated from *S. japonicus* confirmed the presence of the retained intron.

To create temperature sensitive mutants of *cdc25-D9*, a PCR-based random mutagenesis strategy was adapted from [S3] with some modifications. An adaptor strain that carries the C-terminally truncated *ura4* ORF cassette at the 3'UTR region of *cdc25* locus was generated using the plasmid pKanMX6-Ura4ΔC *S. japonicus*. Error-prone PCR was performed using the template plasmid pKS-Ura4ΔC *S. japonicus* that harbours *cdc25* ORF to be mutagenized. The adaptor strain was transformed with mutagenised PCR products and transformants were screened at 36°C for temperature-sensitive colonies.

Image acquisition and analysis

Time-lapse confocal images were obtained using Nikon Eclipse Ti-E inverted system equipped with CSU-X1 Spinning Disk Confocal fitted with Andor Ixon3 EM-CCD camera using 100x/1.4NA objective lens. Epifluorescence images were obtained with Zeiss Axio Observer Z1 microscope fitted with α Plan-FLUAR 100x/1.45NA objective lens and Hamamatsu Orca-Flash4.0 C11440 camera.

Nuclear circularity index was measured at the centre z-plane of interphase cells using formula $\Theta = 4\mathrm{pi}A/P^2 \sim 12.57A/P^2$, where Θ is

circularity, A is the area and P is the perimeter. Perimeter and area were measured using imageJ software (http://rsb.info.nih.gov/ij/; National Institutes of Health, Bethesda, MD, USA). Nuclear surface area in mitotic cells was measured as described previously [S4]. For interphase measurements we used late G2 cells (11-14 μm length).

Staining of lipid droplets in *S. japonicus* cells using BODIPY 493/503 was performed as in [S5]. Staining of membranes in *S. pombe* cells using DiO6 was performed as in [S6].

Growth rate measurements, cell cycle synchronization and germination of spores

Fission yeast cells grown overnight at 30°C to log-phase were diluted to OD_{595} 0.1. Optical density measurements were performed every hour until the OD_{595} reached approximately 0.6. All experiments were performed in three independent replicates.

S. pombe (*cdc25-22*) and *S. japonicus* (*cdc25-D9*) *cdc25* mutant cells grown overnight at the permissive temperature of 24°C in YES medium to OD_{595} 0.2-0.3 were shifted to 36°C for 3.5 hours. Release from the G2 block was performed by cooling cells in ice-cold water bath to 24°C followed by incubation at 24°C. Synchronization was verified by measuring the numbers of bi-nucleate and septated cells.

Sporulating heterozygous diploids were incubated with 0.5% glusulase (Perkin-Elmer) at 30°C for overnight. After washing with 30% ethanol cell

mixture was checked for the presence of vegetative cells. For selective germination washed spores were inoculated in minimal media lacking uracil and grown at 30°C for 24 hours.

Protein extraction and immunoprecipitation

Cell pellets were snap-frozen in liquid nitrogen and diluted in 100µl volume of IP buffer (50mM Tris-HCl, 150mM NaCl, 1% NP-40, 2 mM EDTA, 50 mM NaF, 0.1 mM Na3VO4, Roche protease inhibitor cocktail). Cell disruption was performed in IP buffer by homogenization with glass beads in a Mini Bead Beater (Biospec, Bartlesville, OK, USA) at 4°C. Total cell lysates were harvested and centrifuged at 16,000g for 10 minutes to remove cell debris. Soluble fractions were adjusted to the same total protein concentration, diluted with IP buffer excluding NP-40 and incubated with GFP-Trap beads (ChromoTek, Munich, Germany) for 2 hours at 4°C. Beads were washed 3 times with 1 ml of IP (excluding NP-40) and resuspended in SDS-loading buffer.

For phosphatase treatment experiments, proteins were extracted as above. Protein extracts were then incubated with GFP-trap beads, washed three times with buffer (50mM Tris-HCl, 150mM NaCl and Roche protease inhibitor cocktail) and subjected to phosphatase treatment on beads using either calf intestinal phosphatase (CIP; NEB) or the lambda phosphatase (λ, NEB) followed by resuspension in SDS-loading buffer.

Protein samples were subjected to SDS-PAGE and standard Western blotting. Ned1-GFP was probed by mouse *anti*-GFP antibody (Roche); mouse *anti-a*-tubulin antibody (kindly provided by K. Gould) served to monitor sample loading. For chemiluminescence detection we used FUSION Solo (secondary antibodies were anti-mouse-HRP (GE healthcare) followed by development with Clarity ECL substrate (Bio-rad). For some experiments, we used the Odyssey Infrared Imaging System (LI-COR Biosciences) with IRDye800 conjugated *anti*-mouse antibodies.

For Phos-tag assays, 6% acrylamide resolving gels containing 12,5 μM Phos-tag (Wako Pure Chemical Industries) and 12,5 μM $MnCl_2$ without SDS were prepared freshly for each experiment [S7]. To strengthen low percentage gels containing Phos-tag we increased amount of ammonium persulfate to 0.2%. Electrophoresis was carried out at constant current of 20mA for 3 hours.

S. japonicus Ned1 ORF was cloned into pMal-c5x vector (NEB) for expression as a maltose binding protein (MBP) fusion. Protein expression was induced in *Escherichia coli* Rosetta2 strain overnight, using 0.4 mM IPTG at 18°C. Proteins were purified on amylose beads (NEB) according to the manufacturer's protocol.

Mass spectrometry (MS) analysis

Isolated proteins were denatured, reduced with Tris 2-carboxyethyl phosphine, alkylated with iodoacetamide, and digested overnight at 37˚C with

Trypsin Gold (Promega) or Chymotrypsin (Princeton Separations) after diluting to 2M urea with 50 mM Tris pH 8.5. The resulting peptides were subjected to 2D LC-MS/MS (MudPIT) on a Thermo LTQ as previously detailed [S8]. Thermo RAW files were converted to DTA files using Scansifter [S9] and spectra with fewer than 20 peaks were excluded from analysis. Spectra were searched against the *S. pombe* database (pombase.org, May 2011) using the SEQUEST (TurboSequest v.27 rev12) algorithm on a high performance computing cluster (Advanced Computing Center for Research & Education at Vanderbilt University). We added contaminant proteins (e.g. keratin, IgG) to the complete *S. pombe* database and reversed and concatenated all sequences to allow estimation of false discovery rates (10186 total entries). Variable modifications (C+57, M+16, [STY]+80 for all spectra), strict trypsin cleavage, <10 missed cleavages, fragment mass tolerance: 0.00 Da (because of rounding in SEQUEST, this results in 0.5 Da tolerance), and parent mass tolerance: 2.5 Da were allowed. Peptide identifications were assembled and filtered in Scaffold (v4.1.1, Proteome Software, Portland, OR) using the following criteria: minimum of 99% protein identification probability; minimum of 5 unique peptides; minimum of 90% peptide identification probability; minimum peptide length of five amino acids; minimum number of one tryptic terminus. Scaffold PTM was used to filter the spectra and analyze phosphorylation sites (v2.1.3, Proteome Software, Portland, OR). Phosphorylation sites are reported with their site localization scores and Ascores [S10].

***In vitro* kinase assay**

Immunoprecipitated wild type or mutated Ned1-GFP was washed three times with protein kinase buffer (10 mM Tris, pH 7.4, 10 mM MgCl2, and 1 mM DTT), and then resuspended in kinase buffer supplemented with 10 μM cold ATP, 5 μCi of [^{32}P]ATP, and 100 ng of purified insect cell–produced Cdc2-Cdc13 in a total volume of 20 μl. The reactions were incubated at 30°C for 30 minutes before being terminated by the addition of SDS sample buffer. Proteins were separated by 4-12% NuPAGE Bis-Tris Precast Gels (Life Technologies, Grand Island, NY), transferred to polyvinylidene fluoride (PVDF) membrane, and phosphorylated proteins were visualized by autoradiography. The same membrane was wetted with methanol and blotted with anti-GFP antibody (Roche, Indianapolis, IN) to visualize the level of proteins used in the kinase assays. MBP-Ned1$^{S.\ japonicus}$ and MBP proteins were incubated with Cdc2-Cdc13 complexes and separated on SDS-PAGE, followed by staining with Coomassie blue G250 to visualize the proteins. The protein gel was then dried in a gel dryer, followed by exposure to film to examine phosphorylation.

Supplemental References

S1. Larkin, M.A., Blackshields, G., Brown, N.P., Chenna, R., McGettigan, P.A., McWilliam, H., Valentin, F., Wallace, I.M., Wilm, A., Lopez, R., et al. (2007). Clustal W and Clustal X version 2.0. Bioinformatics *23*, 2947-2948.

S2. Rhind, N., Chen, Z., Yassour, M., Thompson, D.A., Haas, B.J., Habib, N., Wapinski, I., Roy, S., Lin, M.F., Heiman, D.I., et al. (2011). Comparative functional genomics of the fission yeasts. Science *332*, 930-936.

S3. Tang, X., Huang, J., Padmanabhan, A., Bakka, K., Bao, Y., Tan, B.Y., Cande, W.Z., and Balasubramanian, M.K. (2011). Marker reconstitution mutagenesis: a simple and efficient reverse genetic approach. Yeast *28*, 205-212.

S4. Yam, C., He, Y., Zhang, D., Chiam, K.H., and Oliferenko, S. (2011). Divergent strategies for controlling the nuclear membrane satisfy geometric constraints during nuclear division. Current biology : CB *21*, 1314-1319.
S5. He, Y., Yam, C., Pomraning, K., Chin, J.S., Yew, J.Y., Freitag, M., and Oliferenko, S. (2014). Increase in cellular triacylglycerol content and emergence of large ER-associated lipid droplets in the absence of CDP-DG synthase function. Molecular biology of the cell *25*, 4083-4095.
S6. Koning, A.J., Lum, P.Y., Williams, J.M., and Wright, R. (1993). DiOC6 staining reveals organelle structure and dynamics in living yeast cells. Cell motility and the cytoskeleton *25*, 111-128.
S7. Kinoshita, E., Kinoshita-Kikuta, E., Takiyama, K., and Koike, T. (2006). Phosphate-binding tag, a new tool to visualize phosphorylated proteins. Molecular & cellular proteomics : MCP *5*, 749-757.
S8. Roberts-Galbraith, R.H., Chen, J.S., Wang, J., and Gould, K.L. (2009). The SH3 domains of two PCH family members cooperate in assembly of the Schizosaccharomyces pombe contractile ring. The Journal of cell biology *184*, 113-127.
S9. Ma, Z.Q., Tabb, D.L., Burden, J., Chambers, M.C., Cox, M.B., Cantrell, M.J., Ham, A.J., Litton, M.D., Oreto, M.R., Schultz, W.C., et al. (2011). Supporting tool suite for production proteomics. Bioinformatics *27*, 3214-3215.
S10. Beausoleil, S.A., Villen, J., Gerber, S.A., Rush, J., and Gygi, S.P. (2006). A probability-based approach for high-throughput protein phosphorylation analysis and site localization. Nature biotechnology *24*, 1285-1292.
