# Supplementary material for: Temporal Regulation of Lipin Activity Diverged to Account for Differences in Mitotic Programs
Source: Curr Biol. 2016 Jan 25;26(2):237–43. doi: 10.1016/j.cub.2015.11.061 (PMC4728079; doi:10.1016/j.cub.2015.11.061)
Supplement: Document S2. Article plus Supplemental Information [file mmc2.pdf]

Report

Current Biology

Temporal Regulation of Lipin Activity Diverged to Account for Differences in Mitotic Programs

Highlights

- Lipin phosphorylation by CDK drives mitotic nuclear envelope expansion in *S. pombe*
- CDK-dependent lipin phosphorylation is required for closed mitosis
- Lipin is not regulated by mitotic CDK in the related species *S. japonicus*
- Interspecies gene swaps reveal species-specific trans-regulation of lipin activity

Authors

Maria Makarova, Ying Gu, Jun-Song Chen, Janel Renée Beckley, Kathleen Louise Gould, Snezhana Oliferenko

Correspondence

snezhana.oliferenko@kcl.ac.uk

In Brief

Using closely related yeasts, Makarova et al. uncover a molecular basis for variability in nuclear envelope expansion during mitosis. They show that cells undergoing closed mitosis expand their nuclear envelope prior to division by entraining inactivation of the phosphatidic acid flux regulator lipin to high CDK activity.

CrossMark

Makarova et al., 2016, Current Biology *26*, 237–243
January 25, 2016 ©2016 The Authors
http://dx.doi.org/10.1016/j.cub.2015.11.061

CellPress

Current Biology
Report

Temporal Regulation of Lipin Activity Diverged to Account for Differences in Mitotic Programs

Maria Makarova,[1] Ying Gu,[1] Jun-Song Chen,[2] Janel Renée Beckley,[2] Kathleen Louise Gould,[2] and Snezhana Oliferenko[1,*]
[1]Randall Division of Cell and Molecular Biophysics, King's College London, London SE1 1UL, UK
[2]Department of Cell and Developmental Biology, Vanderbilt University, Nashville, TN 37240, USA
*Correspondence: snezhana.oliferenko@kcl.ac.uk
http://dx.doi.org/10.1016/j.cub.2015.11.061
This is an open access article under the CC BY license (http://creativecommons.org/licenses/by/4.0/).

SUMMARY

Eukaryotes remodel the nucleus during mitosis using a variety of mechanisms that differ in the timing and the extent of nuclear envelope (NE) breakdown. Here, we probe the principles enabling this functional diversity by exploiting the natural divergence in NE management strategies between the related fission yeasts *Schizosaccharomyces pombe* and *Schizosaccharomyces japonicus* [1–3]. We show that inactivation of Ned1, the phosphatidic acid phosphatase of the lipin family, by CDK phosphorylation is both necessary and sufficient to promote NE expansion required for "closed" mitosis in *S. pombe*. In contrast, Ned1 is not regulated during division in *S. japonicus*, thus limiting membrane availability and necessitating NE breakage. Interspecies gene swaps result in phenotypically normal divisions with the *S. japonicus* lipin acquiring an *S. pombe*-like mitotic phosphorylation pattern. Our results provide experimental evidence for the mitotic regulation of phosphatidic acid flux and suggest that the regulatory networks governing lipin activity diverged in evolution to give rise to strikingly dissimilar mitotic programs.

RESULTS AND DISCUSSION

The surface area of a mother nucleus undergoing closed mitosis must increase to allow intranuclear mitotic spindle elongation and formation of the daughter nuclei. The model yeasts *S. pombe* and *Saccharomyces cerevisiae* solve this problem through nuclear envelope (NE) expansion at mitotic entry [1, 4–9]. In contrast, *S. japonicus*, an *S. pombe* relative, does not expand its NE and instead relies on NE breakdown during anaphase to allow chromosome segregation [1, 3].

Abnormal NE proliferation in interphase has been linked to changes in phosphatidic acid metabolism [10–12], suggesting that cell-cycle-dependent mechanisms may similarly regulate NE expansion during mitosis. The phosphatidic acid phosphatase lipin converts phosphatidic acid into diacylglycerol (DAG), which can then be used for production of storage lipids [13] (Figure 1A). When lipin is inactivated in budding yeast, the rate of phospholipid biosynthesis increases and the entire endomembrane system including the NE and the ER expands dramatically [10]. Lipin is regulated negatively by several kinases including Pho85p-Pho80p, Cdc28-CyclinB, PKA, and TORC1 [14–20] and positively by the ER-localized phosphatase Spo7-Nem1 [12, 17, 21] (Figure 1A).

Lack of the lipin Ned1 or its activator Spo7-Nem1 resulted in steady-state expansion of the entire ER in both *S. pombe* and *S. japonicus*, as visualized by the luminal ER marker GFP-ADEL (Figures 1B and 1C, upper panels; see also [22]). Importantly, the interphase nuclei deviated from their normal spherical shape in the lipin pathway mutants of both species (Figures 1B and 1C, lower panels; see Figure S1A for the nuclear pore marker Nup85-GFP). The nuclei of *S. japonicus* cells lacking Ned1, Spo7, or Nem1 exhibited particularly pronounced flares and low circularity indices (Figures 1C and S1A; see the Supplemental Experimental Procedures for image analysis details). In spite of nuclear membrane expansion in *ned1Δ S. japonicus* cells, the timing of NE breakdown did not change (Figure S1B). Microscopic examination of *ned1Δ S. pombe* and *S. japonicus* cells co-expressing the nucleoplasmic marker Pus1-GFP and the mCherry-tagged nucleolar proteins (Bop1 and Erb1, respectively) showed that, unlike in budding yeast [23], the NE flares in the two fission yeast species were not strictly associated with the nucleolus (Figure S1C). The catalytic mutants of Ned1 (Ned1$^{D383E/D385E}$ in *S. pombe* and Ned1$^{D422E/D424E}$ in *S. japonicus*) exhibited comparable ER expansion and formation of NE flares (Figure S1D), suggesting that the observed endomembrane proliferation was due to a lack of Ned1 enzymatic activity [24]. The growth rates of *ned1Δ* strains decreased in both species as compared to the wild-type controls, with *S. japonicus* being more sensitive to the loss of Ned1 (Figure S1E). Taken together, our results suggest that the general logic of the Ned1-centered circuitry is conserved between the two fission yeast species.

Lipin phosphorylation negatively influences its enzymatic activity [25]. To evaluate Ned1 phosphorylation status in both fission yeasts, we performed immunoprecipitation of the GFP-tagged Ned1 proteins from log-phase cultures of *S. pombe* and *S. japonicus* and analyzed their electrophoretic mobility before and after treatment with protein phosphatase. Consistent with a previous report [22], Ned1-GFP isolated from wild-type *S. pombe* migrated as several bands that collapsed into a faster migrating product upon phosphatase treatment (Figure S2A). Under the same conditions, *S. japonicus* Ned1-GFP showed no detectable electrophoretic mobility shift after treatment with

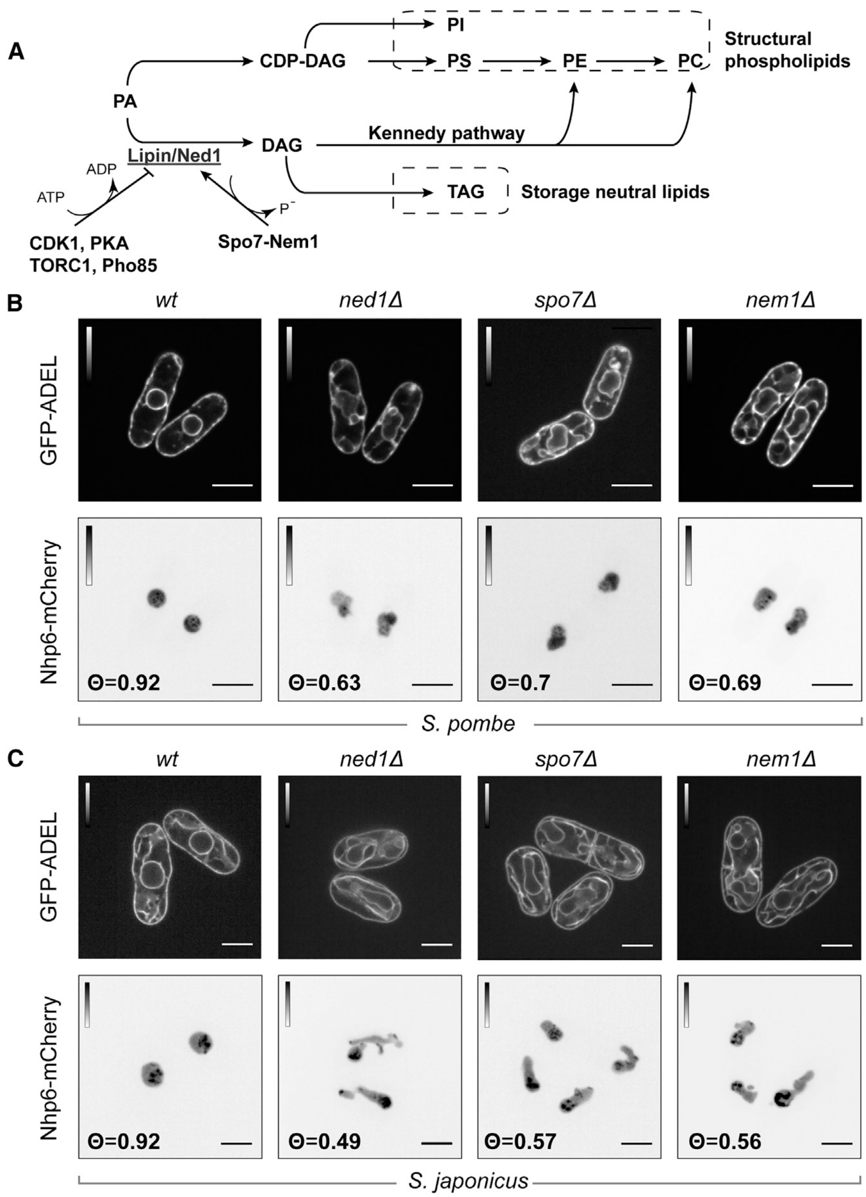

Figure 1. Lipin Dysfunction Leads to NE and ER Expansion Both in *S. pombe* and *S. japonicus*

(A) A schematic diagram representing lipid biosynthesis pathways downstream of phosphatidic acid. CDP-DAG, cytidine diphosphate diacylglycerol; DAG, diacylglycerol; PA, phosphatidic acid; PC, phosphatidylcholine; PE, phosphatidylethanolamine; PI, phosphatidylinositol; PS, phosphatidylserine; TAG, triacylglycerol.

(B and C) Fluorescence confocal images of *S. pombe* (B) and *S. japonicus* (C) cells of the indicated genotypes co-expressing the ER luminal marker GFP-ADEL and the high mobility group protein Nhp6-mCherry marking nucleoplasm. The Nhp6-mCherry images are inverted. Nuclear circularity indices are presented as Θ values (for both *S. pombe* and *S. japonicus*: WT, n = 51 cells; *ned1Δ*, n = 50 cells; *spo7Δ*, n = 50 cells; and *nem1Δ*, n = 50 cells).

The scale bars represent 5 μm. See also Figure S1.

phosphatase (Figure S2A). Yet, Ned1-GFP purified from *spo7Δ* mutants of both *S. pombe* and *S. japonicus* was hyperphosphorylated (Figure S2A), suggesting potential phosphoregulation in both species.

To track changes in Ned1 phosphorylation during the cell cycle, we drove *S. pombe* and *S. japonicus* cells through synchronous mitosis using temperature-sensitive alleles of *cdc25*, a gene controlling the transition from G2 to mitosis [26]. Ned1-GFP was isolated and analyzed by western blotting from cells that were blocked at the G2/M boundary by incubation at the restrictive temperature of 36°C or released into mitosis by decreasing the temperature to 24°C (Figures 2A and S2B). In *S. pombe*, slower migrating forms of Ned1-GFP peaked in mitosis, suggesting that Ned1 was hyperphosphorylated at this stage of the cell cycle (Figure 2A, top panel). Under the same conditions, the electrophoretic migration of *S. japonicus* Ned1-GFP did not change (Figure 2A, bottom panel). To probe mitosis-specific phosphorylation changes further, we performed western blotting of Ned1-GFP isolated from G2-arrested and mitotic extracts in the presence of Phos-tag that allows efficient separation of phosphorylated protein forms [27]. As expected, the *S. pombe* Ned1-GFP exhibited markedly different gel mobility patterns in G2 and mitosis (Figure 2B, left panel). Using this technique, we observed the presence of phosphorylated lipin species in *S. japonicus*. However, the ratio of phosphorylated to non-phosphorylated forms remained comparable between interphase and mitosis (Figure 2B, right panel).

To identify phosphorylation sites on lipin proteins in *S. pombe* and *S. japonicus*, we purified Ned1-GFP from asynchronously growing cultures and performed 2D-liquid chromatography-tandem mass spectrometry (LC-MS/MS). We identified two clusters of phosphorylation in *S. pombe* Ned1 located in its C terminus—S587/S597/T598/S599 and S627/S629. Notably, S627/S629 represents a repeated relaxed S-P consensus motif for phosphorylation by the cyclin-dependent kinase 1 (CDK1). Analysis of Ned1 of *S. japonicus* revealed two possible phosphorylation sites—S235 located between the N-lipin and catalytic domains and S651 at its C terminus (Figures 2C and S2C).

Because *S. pombe* Ned1 is hyperphosphorylated in mitosis, we sought to determine whether CDK1 is directly involved in its phosphoregulation. Ned1-GFP was purified from *S. pombe* cells arrested at the G2/M boundary and subjected to an in vitro CDK1 kinase assay. Autoradiography revealed that ^{32}P was readily incorporated into the protein (Figure 2D). Confirming our phosphomapping results, mutating S627/S629 residues

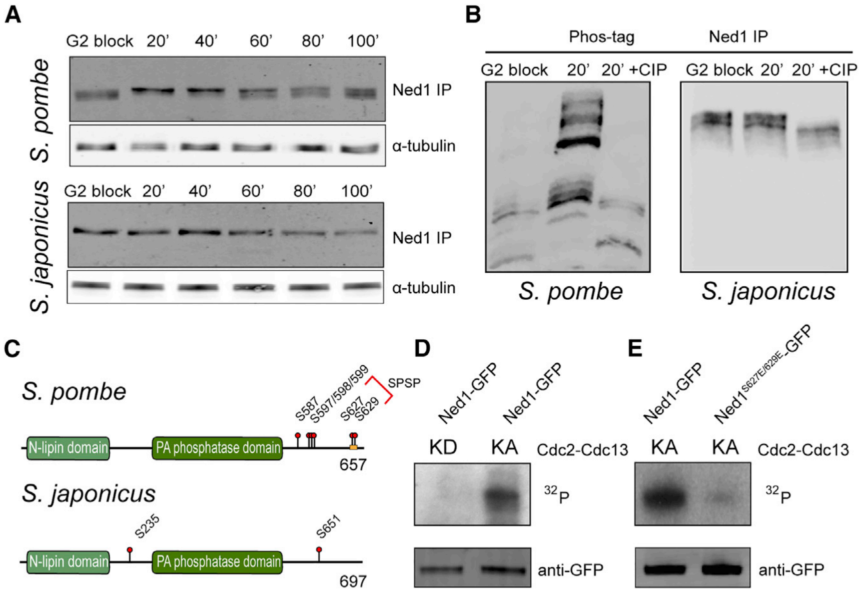

Figure 2. The Lipin Ned1 Is Hyperphosphorylated During Mitosis in *S. pombe*, but Not in *S. japonicus*

(A) Cell-cycle synchronization experiments utilizing the temperature-sensitive mutant alleles of *cdc25* (*cdc25-22* for *S. pombe* and *cdc25-D9* for *S. japonicus*). Time after release from the G2/M block is in minutes. Ned1-GFP from each time point was immunoprecipitated and subjected to WB analysis. WB for α-tubulin of input lysates was used as a loading control.

(B) Western blot analysis of immunoprecipitated Ned1-GFP from G2/M-blocked and mitotic cells (20 min after release) of *S. pombe* and *S. japonicus*. Samples were separated on 6% SDS-PAGE gel in the presence of Phos-tag. The mitotic samples were also subjected to CIP treatment.

(C) Schematic diagrams representing Ned1 S/T phosphorylation sites identified by LC-MS/MS analysis in *S. pombe* and *S. japonicus*. Positions of the evolutionarily conserved N-lipin and catalytic domains are also shown. The red bracket indicates a putative CDK1 phosphorylation site.

(D) CDK1 phosphorylates the *S. pombe* lipin Ned1 in vitro. Cdc2-Cdc13 kinase assays were performed using Ned1-GFP purified from G2-arrested *cdc25-22 S. pombe* cells. Ned1-GFP was incubated either with active (KA) or inactive (KD) Cdc2-Cdc13 kinase complexes. Half of the kinase reaction was used to detect phosphorylation by autoradiography (^{32}P) and half was used in western blots with anti-GFP antibodies.

(E) Serine residues at positions 627 and 629 are essential for phosphorylation of Ned1 by Cdc2-Cdc13 in vitro. The kinase assays were performed using either wild-type or S627E/S629E mutant Ned1 proteins purified from *cdc25-22 S. pombe* cells arrested at G2/M transition.

See also Figure S2.

to phosphomimetic glutamic acid (Ned1$^{S627E/S629E}$) virtually abolished CDK phosphorylation in vitro (Figure 2E). These data raise the possibility that the lipin proteins are differentially regulated in the two fission yeast species, with the *S. pombe* ortholog undergoing mitotic phosphorylation by CDK1.

To understand the functional consequences of mitotic phosphorylation of Ned1 in *S. pombe*, we mutated the sequence encoding the CDK consensus residues S627/S629 to either alanine or the phosphomimetic glutamic acid at the endogenous locus. *S. pombe* cells solely expressing the phosphomimetic Ned1$^{S627E/S629E}$ variant exhibited expansion of the ER and the misshapen nuclei, indicative of a loss-of-function phenotype (Figure 3A). The nuclear surface area in interphase mutant cells was approximately 34% ± 3% more than the control (n = 10). Because the transient mitotic phosphorylation of Ned1 coincides with an increase in the nuclear surface area in wild-type *S. pombe*, we wondered whether the mutant cells expressing phosphomimetic Ned1 failed to expand the NE further during mitosis. Indeed, unlike in the wild-type cells where the NE surface area grew by 33% ± 4% during mitosis (n = 20 cells; see also [1]), the Ned1$^{S627E/S629E}$ mutant cells showed only a modest 12% ± 5% increase (n = 20; Figures 3B and 3C). We obtained comparable results using *spo7Δ* cells where Ned1 remained hyperphosphorylated (Figure S3A; n = 10). Thus, constitutive phosphorylation of Ned1 on CDK sites in *S. pombe* leads to the steady-state expansion of the NE rather than restricting this process specifically to mitosis.

We repeatedly failed to obtain a haploid strain where Ned1 was refractory to phosphorylation by CDK (Ned1$^{S627A/S629A}$), suggesting that the mutant protein did not support growth. To investigate this possibility, we constructed a diploid strain where one of the wild-type copies of *ned1* was replaced by the *ned1*$^{S627A/S629A}$ allele tagged with the selectable auxotrophic marker *ura4+*. The growth rates of diploid *WT/WT* and *WT/ned1*$^{S627A/S629A}$ cells at 30°C were comparable although the *WT/ned1*$^{S627A/S629A}$ diploids exhibited pronounced lag phase at lower cell densities (Figure S3B). Consistent with the possibility of higher lipin activity leading to accumulation of neutral lipids [28], we observed an increase in lipid droplet abundance in hemizygous diploids (Figure S3C). After induction of sporulation, spores carrying the *ned1*$^{S627A/S629A}$ mutant allele were germinated in the absence of uracil. Interestingly, the S627A/S629A mutation caused a highly penetrant mitotic failure manifested as a so-called "cut" phenotype with the division septum bisecting unsegregated chromosomes (Figure 3D; n = 300). As visualized by membrane staining with vital lipophilic fluorescent dye DiOC6 [29], nuclei of S627A/S629A mutant cells initiated anaphase elongation but eventually collapsed and failed to divide (Figure 3E; n = 10). Thus, CDK1-mediated phosphorylation of Ned1 is required for nuclear division in *S. pombe*.

Mutation of two phosphorylation sites identified in *S. japonicus* Ned1 to alanine (Ned1$^{S235A/S651A}$) did not visibly alter NE and ER morphology, with cells undergoing normal mitotic divisions and maintaining viability (Figure S3D). Consistent with higher lipin activity due to its constitutive dephosphorylation, we observed an increase in the number and the size of lipid droplets in Ned1$^{S235A/S651A}$ mutant cells (Figure S3E). On the other hand, *S. japonicus* cells carrying the phosphomimetic Ned1$^{S235E/S651E}$ variant showed NE flares and ER membrane proliferation, somewhat similar to *ned1Δ* mutant (Figure S3D). The mutant cells also exhibited fewer lipid droplets as compared to the control (Figure S3E). These results suggest that, although *S. japonicus*

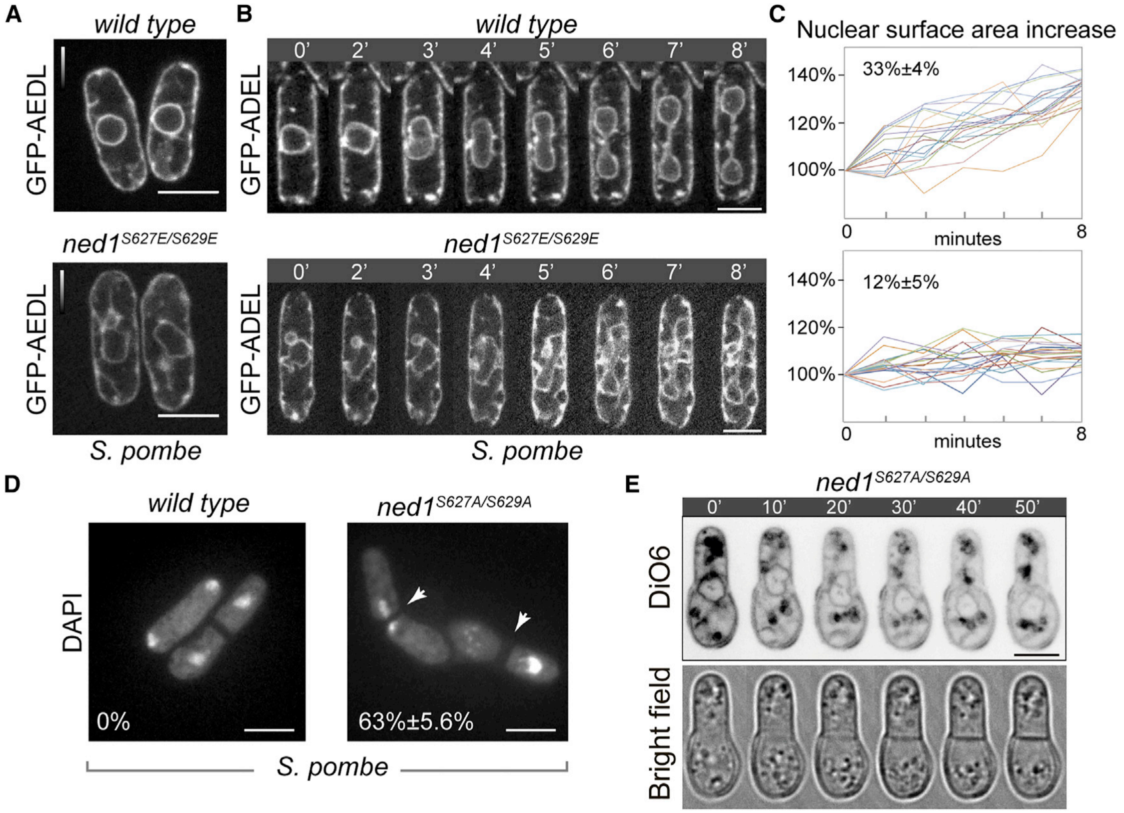

Figure 3. Phosphorylation of the Lipin Ned1 by CDK1 Is Required for Nuclear Division in *S. pombe*

(A) Single z-plane confocal images of *S. pombe* expressing the ER marker GFP-ADEL in the wild-type or phosphomimetic Ned1$^{S627E/S629E}$ mutant background.

(B) Time-lapse sequences of the wild-type (top) and Ned1$^{S627E/S629E}$ (bottom) cells undergoing mitosis. The ER is marked by GFP-ADEL. Time is in minutes.

(C) Plots representing experimentally determined values of the nuclear surface area during nuclear division in the wild-type (top; n = 20) and Ned1$^{S627E/S629E}$ mutant (bottom; n = 20) *S. pombe* cells.

(D) Epifluorescence images of fixed and DAPI-stained *S. pombe* cells of the indicated genotypes, where both wild-type and the mutant Ned1 proteins are tagged with GFP. Shown are post-mitotic cells originating from sporulation of the heterozygous diploids carrying either *ned1-gfp* or *ned1*$^{S627A/S629A}$*-gfp* integrated at the native *ned1* locus. Arrows indicate cells with "cut" phenotype. The percentage of cells exhibiting a "cut" phenotype is shown (n = 300 cells).

(E) Time-lapse sequence of a Ned1$^{S627A/S629A}$ cell undergoing first mitosis after spore germination. Membranes including the NE and the ER are marked by a vital dye DiO6 (top). The phase-contrast image is shown (bottom). Time is in minutes.

The scale bars represent 5 μm (A, B, D, and E). See also Figure S3.

Ned1 functions in lipid droplet biogenesis, its activity is not regulated during mitosis.

Interspecies differences in the Ned1 phosphorylation and hence mitotic NE expansion strategies can be due to sequence divergence of the protein itself or its differential regulation by *trans*-acting factors in the two fission yeasts. To distinguish between these possibilities, we interchanged the *ned1* open reading frames (ORFs) of *S. pombe* and *S. japonicus*, leaving the host-specific untranslated gene regions intact. Interestingly, germination of *S. pombe* spores carrying the *ned1*$^{S.\ japonicus}$::*ura4+* (*ned1*$^{S.j.}$) allele as its sole *ned1* copy yielded healthy cells exhibiting normal chromosome partitioning as judged by staining the DNA with DAPI (Figure 4A; n = 300). Live cell imaging of mCherry-ADEL-expressing *S. pombe* cells carrying the transplanted Ned1$^{S.j.}$ confirmed that mitosis was phenotypically normal (Figure 4B). Similarly, in *S. japonicus*, swapping the native Ned1 with its *S. pombe* ortholog Ned1$^{S.\ pombe}$ (*ned1*$^{S.p.}$) did not lead to obvious differences in mitotic nuclear dynamics (Figure S4A).

Indeed, the population-doubling times were comparable between the lipin-swapped and the wild-type cultures (2.34 ± 0.18 versus 2.31 ± 0.11 hr for *S. pombe* wild-type and Ned1$^{S.j.}$ and 1.8 ± 0.22 versus 1.84 ± 0.13 hr for *S. japonicus* wild-type and Ned1$^{S.p.}$). The fact that the two lipins could substitute for each other when expressed in a heterologous system indicates that the regulatory modes rather than the lipin protein properties diverged between the two fission yeasts.

The gel migration patterns of Ned1$^{S.j.}$-GFP were markedly different in G2-arrested and mitotic *S. pombe* cells, indicating that the transplanted Ned1 protein could be subject to *S. pombe*-specific mitotic phosphorylation events (Figures 4C, S4B, and S4C). Recombinant Ned1$^{S.\ japonicus}$ could be phosphorylated by Cdk1 in vitro (Figure S4D), and Ned1$^{S.\ japonicus}$ has several putative CDK phosphorylation sites, including five at its C terminus, S499, S538, T620, T638, and S651, suggesting that similar phosphoregulation could occur in *S. pombe* cells. Replacement of these serine and threonine residues with non-phosphorylatable alanines rendered the

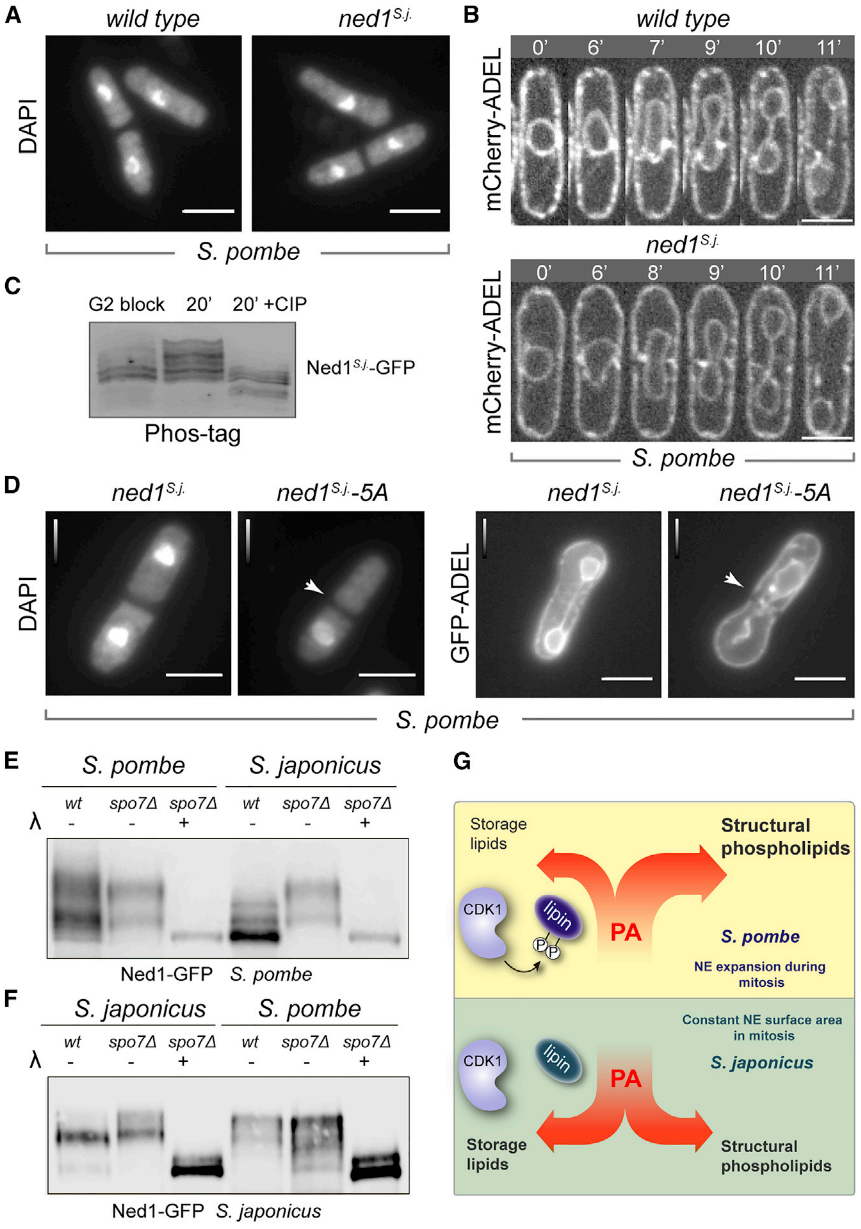

Figure 4. Mitotic Regulation of Ned1 Activity Diverged in the Fission Yeast Clade

(A) Single z-plane epifluorescence images of fixed and DAPI-stained *S. pombe* cells expressing either the wild-type Ned1 or its *S. japonicus* ortholog Ned1$^{S.j.}$ (n = 300 cells). Shown are post-mitotic cells originating from sporulation of the heterozygous diploids carrying either *ned1-gfp* or *ned*$^{S.j.}$*-gfp* integrated at the native *ned1* locus.

(B) Time-lapse confocal sequences of *S. pombe* cells expressing either the "wild type" Ned1-GFP (top) or "transplanted" Ned1$^{S.j.}$-GFP (bottom) proteins as an only source of Ned1. The ER is labeled by mCherry-ADEL. Time is in minutes.

(C) Western blot analysis of immunoprecipitated Ned1$^{S.j.}$-GFP purified from either G2-arrested or mitotic *cdc25-22 S. pombe* cells in the presence of Phos-tag. The mitotic sample was also subjected to CIP treatment.

(D) (Left panels) Single z-plane epifluorescence images of fixed and DAPI-stained *S. pombe* cells expressing either the wild-type *S. japonicus* ortholog Ned1$^{S.j.}$ (left) or the non-phosphorylatable mutant Ned1$^{S.j.}$-5A (right) as an only source of Ned1 protein. Shown are post-mitotic cells originating from sporulation of the heterozygous diploids carrying either *ned*$^{S.j.}$*-gfp* or *ned*$^{S.j.}$*-5A-gfp* integrated at the native *ned1* locus. (Right panels) Single z-plane epifluorescence images of *S. pombe* cells co-expressing the ER marker GFP-ADEL and either Ned1$^{S.j.}$-GFP (left) or the non-phosphorylatable Ned1$^{S.j.}$-5A-GFP mutant (right) as an only source of Ned1 protein are shown. The experiment was performed as above.

(E) Western blot analysis of immunoprecipitated Ned1$^{S.p.}$;-GFP purified from *S. pombe* and *S. japonicus* cells of the indicated genotypes in the presence of Phos-tag. The *spo7Δ* samples were also subjected to λ phosphatase treatment.

(F) Western blot analysis of immunoprecipitated Ned1$^{S.j.}$-GFP purified from *S. japonicus* and *S. pombe* cells of the indicated genotypes in the presence of Phos-tag. The *spo7Δ* samples were also subjected to λ phosphatase treatment.

(G) A pictorial model summarizing our current hypothesis on how the NE surface area is controlled during mitosis in the related fission yeast species.

The scale bars represent 5 μm (A, B, and D). See also Figure S4.

transplanted Ned1$^{S.j.}$ defective in supporting mitotic division in *S. pombe* cells. Similar to the phenotype observed in *S. pombe* *ned1*$^{S627A/S629A}$ mutant (Figure 3D), many germinated spores carrying *ned1*$^{S.j.}$*-5A*::*ura4+* allele failed to properly partition chromosomes and divide the nucleus in the first mitotic division (Figure 4D; 43% ± 7% cells exhibited "cut" phenotype; n = 300). On the other hand, the phosphomimetic variant Ned1$^{S.j.}$-5E caused a loss-of-function phenotype in *S. pombe*, with mutant cells exhibiting constitutive ER and nuclear membrane expansion (Figure S4E). When mutated in *S. japonicus*, the same phosphomimetic mutant triggered ER-NE expansion during interphase and decreased lipid droplet formation (Figures S4F and S4G). Introduction of the 5A mutant in *S. japonicus* did not cause mitotic abnormalities but produced cells with more lipid droplets (Figures S4F and S4G), reminiscent of the phenotype observed in *ned1*$^{S235A/S651A}$ genetic background (Figure S3E).

To address the potential contribution of Spo7-Nem1 phosphatase to lipin regulation in the two species, we analyzed the electrophoretic migration of Ned1$^{S. pombe}$ in the presence of Phos-tag in *S. pombe* and *S. japonicus* asynchronous wild-type and *spo7*Δ cultures. When expressed in its native wild-type environment, Ned1$^{S. pombe}$ migrated as multiple phospho-forms, suggesting potential phosphoregulation by multiple inputs (Figure 4E). Strikingly, when transplanted in *S. japonicus* cells, Ned1$^{S. pombe}$ acquired a distinct electrophoretic mobility pattern, consistent with the pronounced presence of a dephosphorylated form. Introducing the transplanted protein into the *spo7*Δ background led to increased phosphorylation (Figure 4E). This suggests that the *S. pombe* lipin, normally a highly phosphorylated

protein, is subject to massive dephosphorylation by Spo7-Nem1 phosphatase in *S. japonicus*.

In a reciprocal experiment, we analyzed the phospho-state of the *S. japonicus* lipin in both yeasts. We consistently detected a major phospho-form together with a dephosphorylated species of lipin in *S. japonicus* wild-type cells. As expected, the lack of Spo7 led to hyperphosphorylation of Ned1 (Figure 4F). When transplanted into *S. pombe*, Ned1$^{S.\ japonicus}$ migrated differently than it did in *S. japonicus,* consistent with differential phosphoregulation in this species (Figure 4F).

Taken together, our results suggest that the fundamental differences between mitotic strategies of the two fission yeasts might depend on divergence of the regulatory networks controlling lipin activity. Phosphorylation of Ned1 by CDK1 in *S. pombe* allows this organism to inactivate the bulk of the enzyme specifically in mitosis. Lipin inactivation presumably triggers increased phospholipid production required for massive NE expansion during "closed" nuclear division. On the other hand, overall phosphorylation status of lipin in *S. japonicus* remains constant throughout the cell cycle (Figure 4G). This does not mean that lipin activity is not regulated by phosphorylation in this organism (see Figures 2B, 2C, S3D, and S3E)—only that such phosphoregulation is not entrained to the mitotic cell cycle. Indeed, lipin activity must be dynamically controlled within a cell to produce DAG in a spatially restricted manner, supporting lipid droplet biogenesis [30], vacuole homeostasis [31], and other biosynthetic and signaling events [25]. At steady state, the overall lipin phosphorylation and, hence, its activation status may reflect the opposing kinase and phosphatase activities. Mitotic transition to the predominantly phosphorylated form of lipin in *S. pombe* could be due to a disruption in this balance, for instance because of concurrent inactivation of the Spo7-Nem1 phosphatase. The fact that the transplanted *S. pombe* lipin is more dephosphorylated in *S. japonicus* suggests a powerful contribution of phosphatase in this species (Figure 4E). Alternatively, it is also possible that pre-existing protein phosphorylation could prevent CDK-dependent modifications of lipin [32].

As compared to *S. pombe*, lipin deficiency in *S. japonicus* produces considerably stronger expansion of the NE-ER system and has a more deleterious impact on cellular growth rate (Figures 1 and S1). Different outputs downstream of lipin inactivation may necessitate keeping the bulk of lipin relatively active at all times in cycling *S. japonicus* cells. Work in budding yeast uncovered crosstalk between lipin function and transcriptional control of a cohort of inositol-responsive lipid biosynthesis genes [25], but this gene regulation circuitry is not conserved between budding and fission yeasts. Mammalian lipins have been also implicated in transcriptional regulation of genes involved in fatty acid oxidation and adipocyte differentiation [33]. Determining relative contributions of the lipin enzymatic and gene regulation modalities in both *S. pombe* and *S. japonicus* and deducing species-specific response patterns to genetic perturbations of the lipin-centered circuitry will be important to illuminate possible physiological and metabolic foundations for the distinct lipin network topologies within the clade.

Metazoan lipins and the Spo7-Nem1-related phosphatases are important for NE/ER biogenesis and mitotic remodeling [21, 34–37] and function at the intersection of several metabolic pathways [33, 38]. The two fission yeast species with their naturally divergent NE expansion strategies provide an excellent system yielding fundamental insights into lipin function and control of phosphatidic acid flux potentially relevant to all eukaryotes.

EXPERIMENTAL PROCEDURES

Yeast Strains and Culture Conditions

S. pombe and *S. japonicus* strains used in this study and their genotypes are listed in Table S1. Genetic methods, culturing conditions, and transformation procedures for both species have been described previously [39–41]. For details of strain construction, protein biochemistry, and imaging methods, please see the Supplemental Experimental Procedures.

SUPPLEMENTAL INFORMATION

Supplemental Information includes Supplemental Experimental Procedures, four figures, and one table and can be found with this article online at http://dx.doi.org/10.1016/j.cub.2015.11.061.

AUTHOR CONTRIBUTIONS

M.M. did in vivo experiments and co-wrote the manuscript. Y.G. generated a number of *S. japonicus* strains including several fluorescent tags, a conditional *cdc25* mutant, and a *ned1* replacement strain. J.-S.C. performed in vitro kinase assays and mass spectrometry. J.R.B. analyzed mass spectrometry data and edited the manuscript. K.L.G. provided input into the design and interpretation of experiments and edited the manuscript. S.O. guided the project and co-wrote the manuscript.

ACKNOWLEDGMENTS

We are grateful to G. Jedd, A. Vjestica, D. Zhang, and M. Balasubramanian for discussions throughout this work and to E. Makeyev for suggestions on the manuscript. The work has been supported by NIH grant GM101035 to K.L.G. and the Wellcome Trust Senior Investigator Award (103741/Z/14/Z) to S.O.

Received: July 6, 2015
Revised: October 27, 2015
Accepted: November 17, 2015
Published: January 7, 2016

REFERENCES

1. Yam, C., He, Y., Zhang, D., Chiam, K.H., and Oliferenko, S. (2011). Divergent strategies for controlling the nuclear membrane satisfy geometric constraints during nuclear division. Curr. Biol. *21*, 1314–1319.
2. Aoki, K., Hayashi, H., Furuya, K., Sato, M., Takagi, T., Osumi, M., Kimura, A., and Niki, H. (2011). Breakage of the nuclear envelope by an extending mitotic nucleus occurs during anaphase in Schizosaccharomyces japonicus. Genes Cells *16*, 911–926.
3. Gu, Y., Yam, C., and Oliferenko, S. (2012). Divergence of mitotic strategies in fission yeasts. Nucleus *3*, 220–225.
4. Zheng, L., Schwartz, C., Magidson, V., Khodjakov, A., and Oliferenko, S. (2007). The spindle pole bodies facilitate nuclear envelope division during closed mitosis in fission yeast. PLoS Biol. *5*, e170.
5. Lim H W, G., Huber, G., Torii, Y., Hirata, A., Miller, J., and Sazer, S. (2007). Vesicle-like biomechanics governs important aspects of nuclear geometry in fission yeast. PLoS ONE *2*, e948.
6. Neumann, F.R., and Nurse, P. (2007). Nuclear size control in fission yeast. J. Cell Biol. *179*, 593–600.
7. Witkin, K.L., Chong, Y., Shao, S., Webster, M.T., Lahiri, S., Walters, A.D., Lee, B., Koh, J.L., Prinz, W.A., Andrews, B.J., and Cohen-Fix, O. (2012). The budding yeast nuclear envelope adjacent to the nucleolus serves as a membrane sink during mitotic delay. Curr. Biol. *22*, 1128–1133.

8. Castagnetti, S., Oliferenko, S., and Nurse, P. (2010). Fission yeast cells undergo nuclear division in the absence of spindle microtubules. PLoS Biol. *8*, e1000512.
9. Jorgensen, P., Edgington, N.P., Schneider, B.L., Rupes, I., Tyers, M., and Futcher, B. (2007). The size of the nucleus increases as yeast cells grow. Mol. Biol. Cell *18*, 3523–3532.
10. Santos-Rosa, H., Leung, J., Grimsey, N., Peak-Chew, S., and Siniossoglou, S. (2005). The yeast lipin Smp2 couples phospholipid biosynthesis to nuclear membrane growth. EMBO J. *24*, 1931–1941.
11. Han, G.S., O'Hara, L., Carman, G.M., and Siniossoglou, S. (2008). An unconventional diacylglycerol kinase that regulates phospholipid synthesis and nuclear membrane growth. J. Biol. Chem. *283*, 20433–20442.
12. Siniossoglou, S., Santos-Rosa, H., Rappsilber, J., Mann, M., and Hurt, E. (1998). A novel complex of membrane proteins required for formation of a spherical nucleus. EMBO J. *17*, 6449–6464.
13. Han, G.S., Wu, W.I., and Carman, G.M. (2006). The Saccharomyces cerevisiae Lipin homolog is a Mg2+-dependent phosphatidate phosphatase enzyme. J. Biol. Chem. *281*, 9210–9218.
14. O'Hara, L., Han, G.S., Peak-Chew, S., Grimsey, N., Carman, G.M., and Siniossoglou, S. (2006). Control of phospholipid synthesis by phosphorylation of the yeast lipin Pah1p/Smp2p Mg2+-dependent phosphatidate phosphatase. J. Biol. Chem. *281*, 34537–34548.
15. Choi, H.S., Su, W.M., Han, G.S., Plote, D., Xu, Z., and Carman, G.M. (2012). Pho85p-Pho80p phosphorylation of yeast Pah1p phosphatidate phosphatase regulates its activity, location, abundance, and function in lipid metabolism. J. Biol. Chem. *287*, 11290–11301.
16. Choi, H.S., Su, W.M., Morgan, J.M., Han, G.S., Xu, Z., Karanasios, E., Siniossoglou, S., and Carman, G.M. (2011). Phosphorylation of phosphatidate phosphatase regulates its membrane association and physiological functions in Saccharomyces cerevisiae: identification of SER(602), THR(723), AND SER(744) as the sites phosphorylated by CDC28 (CDK1)-encoded cyclin-dependent kinase. J. Biol. Chem. *286*, 1486–1498.
17. Karanasios, E., Han, G.S., Xu, Z., Carman, G.M., and Siniossoglou, S. (2010). A phosphorylation-regulated amphipathic helix controls the membrane translocation and function of the yeast phosphatidate phosphatase. Proc. Natl. Acad. Sci. USA *107*, 17539–17544.
18. Dubots, E., Cottier, S., Péli-Gulli, M.P., Jaquenoud, M., Bontron, S., Schneiter, R., and De Virgilio, C. (2014). TORC1 regulates Pah1 phosphatidate phosphatase activity via the Nem1/Spo7 protein phosphatase complex. PLoS ONE *9*, e104194.
19. Su, W.M., Han, G.S., Casciano, J., and Carman, G.M. (2012). Protein kinase A-mediated phosphorylation of Pah1p phosphatidate phosphatase functions in conjunction with the Pho85p-Pho80p and Cdc28p-cyclin B kinases to regulate lipid synthesis in yeast. J. Biol. Chem. *287*, 33364–33376.
20. Huffman, T.A., Mothe-Satney, I., and Lawrence, J.C., Jr. (2002). Insulin-stimulated phosphorylation of lipin mediated by the mammalian target of rapamycin. Proc. Natl. Acad. Sci. USA *99*, 1047–1052.
21. Kim, Y., Gentry, M.S., Harris, T.E., Wiley, S.E., Lawrence, J.C., Jr., and Dixon, J.E. (2007). A conserved phosphatase cascade that regulates nuclear membrane biogenesis. Proc. Natl. Acad. Sci. USA *104*, 6596–6601.
22. Tange, Y., Hirata, A., and Niwa, O. (2002). An evolutionarily conserved fission yeast protein, Ned1, implicated in normal nuclear morphology and chromosome stability, interacts with Dis3, Pim1/RCC1 and an essential nucleoporin. J. Cell Sci. *115*, 4375–4385.
23. Campbell, J.L., Lorenz, A., Witkin, K.L., Hays, T., Loidl, J., and Cohen-Fix, O. (2006). Yeast nuclear envelope subdomains with distinct abilities to resist membrane expansion. Mol. Biol. Cell *17*, 1768–1778.
24. Han, G.S., Siniossoglou, S., and Carman, G.M. (2007). The cellular functions of the yeast lipin homolog PAH1p are dependent on its phosphatidate phosphatase activity. J. Biol. Chem. *282*, 37026–37035.
25. Siniossoglou, S. (2009). Lipins, lipids and nuclear envelope structure. Traffic *10*, 1181–1187.
26. Nurse, P., Thuriaux, P., and Nasmyth, K. (1976). Genetic control of the cell division cycle in the fission yeast Schizosaccharomyces pombe. Mol. Gen. Genet. *146*, 167–178.
27. Kinoshita, E., Kinoshita-Kikuta, E., Takiyama, K., and Koike, T. (2006). Phosphate-binding tag, a new tool to visualize phosphorylated proteins. Mol. Cell. Proteomics *5*, 749–757.
28. Karanasios, E., Barbosa, A.D., Sembongi, H., Mari, M., Han, G.S., Reggiori, F., Carman, G.M., and Siniossoglou, S. (2013). Regulation of lipid droplet and membrane biogenesis by the acidic tail of the phosphatidate phosphatase Pah1p. Mol. Biol. Cell *24*, 2124–2133.
29. Koning, A.J., Lum, P.Y., Williams, J.M., and Wright, R. (1993). DiOC6 staining reveals organelle structure and dynamics in living yeast cells. Cell Motil. Cytoskeleton *25*, 111–128.
30. Adeyo, O., Horn, P.J., Lee, S., Binns, D.D., Chandrahas, A., Chapman, K.D., and Goodman, J.M. (2011). The yeast lipin orthologue Pah1p is important for biogenesis of lipid droplets. J. Cell Biol. *192*, 1043–1055.
31. Sasser, T., Qiu, Q.S., Karunakaran, S., Padolina, M., Reyes, A., Flood, B., Smith, S., Gonzales, C., and Fratti, R.A. (2012). Yeast lipin 1 orthologue pah1p regulates vacuole homeostasis and membrane fusion. J. Biol. Chem. *287*, 2221–2236.
32. Su, W.M., Han, G.S., and Carman, G.M. (2014). Cross-talk phosphorylations by protein kinase C and Pho85p-Pho80p protein kinase regulate Pah1p phosphatidate phosphatase abundance in Saccharomyces cerevisiae. J. Biol. Chem. *289*, 18818–18830.
33. Reue, K., and Dwyer, J.R. (2009). Lipin proteins and metabolic homeostasis. J. Lipid Res. *50* (*Suppl*), S109–S114.
34. Gorjánácz, M., and Mattaj, I.W. (2009). Lipin is required for efficient breakdown of the nuclear envelope in Caenorhabditis elegans. J. Cell Sci. *122*, 1963–1969.
35. Golden, A., Liu, J., and Cohen-Fix, O. (2009). Inactivation of the C. elegans lipin homolog leads to ER disorganization and to defects in the breakdown and reassembly of the nuclear envelope. J. Cell Sci. *122*, 1970–1978.
36. Han, S., Bahmanyar, S., Zhang, P., Grishin, N., Oegema, K., Crooke, R., Graham, M., Reue, K., Dixon, J.E., and Goodman, J.M. (2012). Nuclear envelope phosphatase 1-regulatory subunit 1 (formerly TMEM188) is the metazoan Spo7p ortholog and functions in the lipin activation pathway. J. Biol. Chem. *287*, 3123–3137.
37. Bahmanyar, S., Biggs, R., Schuh, A.L., Desai, A., Müller-Reichert, T., Audhya, A., Dixon, J.E., and Oegema, K. (2014). Spatial control of phospholipid flux restricts endoplasmic reticulum sheet formation to allow nuclear envelope breakdown. Genes Dev. *28*, 121–126.
38. Reue, K., and Zhang, P. (2008). The lipin protein family: dual roles in lipid biosynthesis and gene expression. FEBS Lett. *582*, 90–96.
39. Gould, K.L. (2004). Protocols for experimentation with Schizosaccharomyces pombe. Methods *33*, 187–188.
40. Aoki, K., Nakajima, R., Furuya, K., and Niki, H. (2010). Novel episomal vectors and a highly efficient transformation procedure for the fission yeast Schizosaccharomyces japonicus. Yeast *27*, 1049–1060.
41. Furuya, K., and Niki, H. (2009). Isolation of heterothallic haploid and auxotrophic mutants of Schizosaccharomyces japonicus. Yeast *26*, 221–233.

Current Biology
Supplemental Information

Temporal Regulation of Lipin Activity Diverged to Account for Differences in Mitotic Programs

Maria Makarova, Ying Gu, Jun-Song Chen, Janel Renée Beckley, Kathleen Louise Gould, and Snezhana Oliferenko

Supplemental Figures

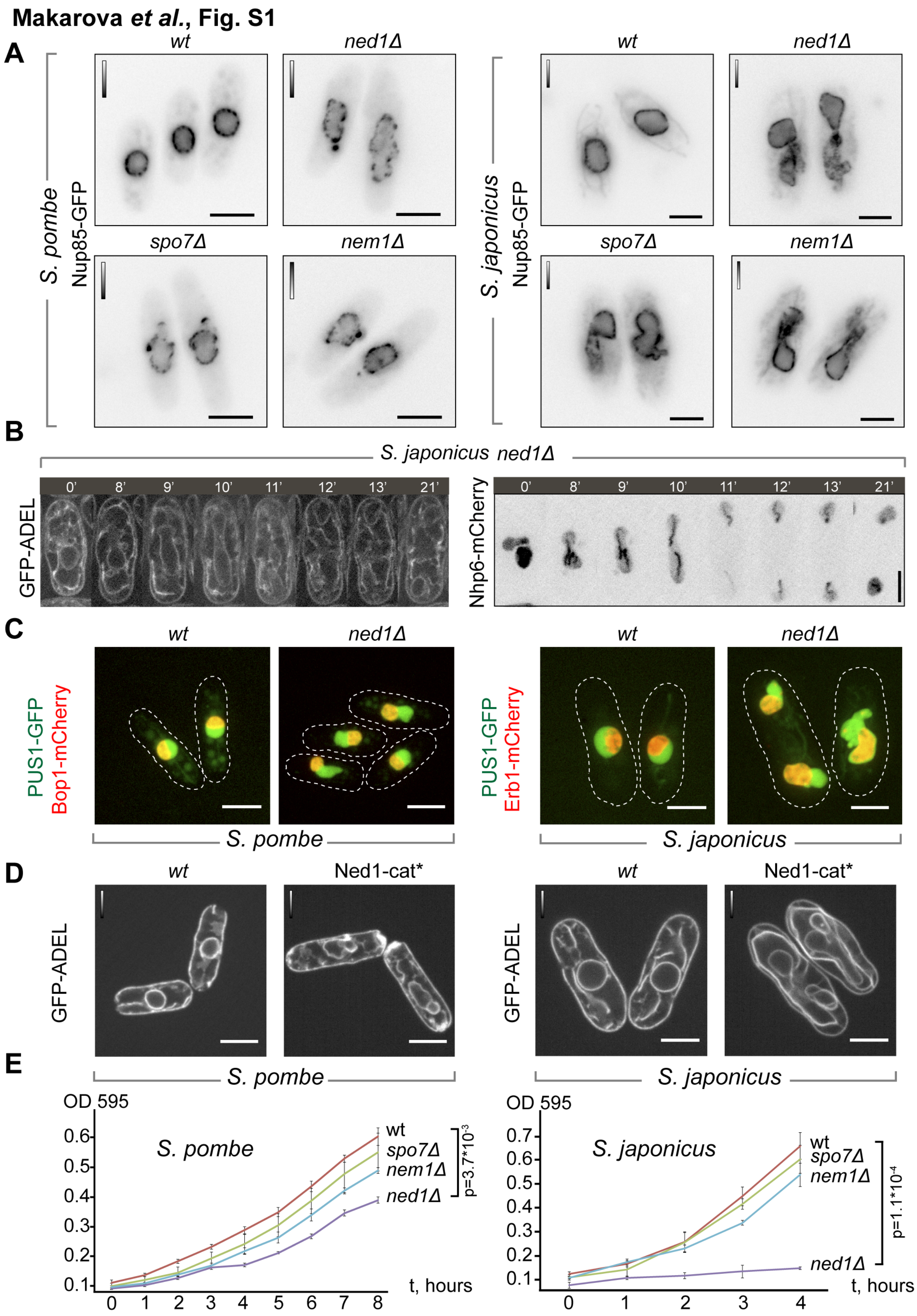

Makarova *et al.*, Fig. S2

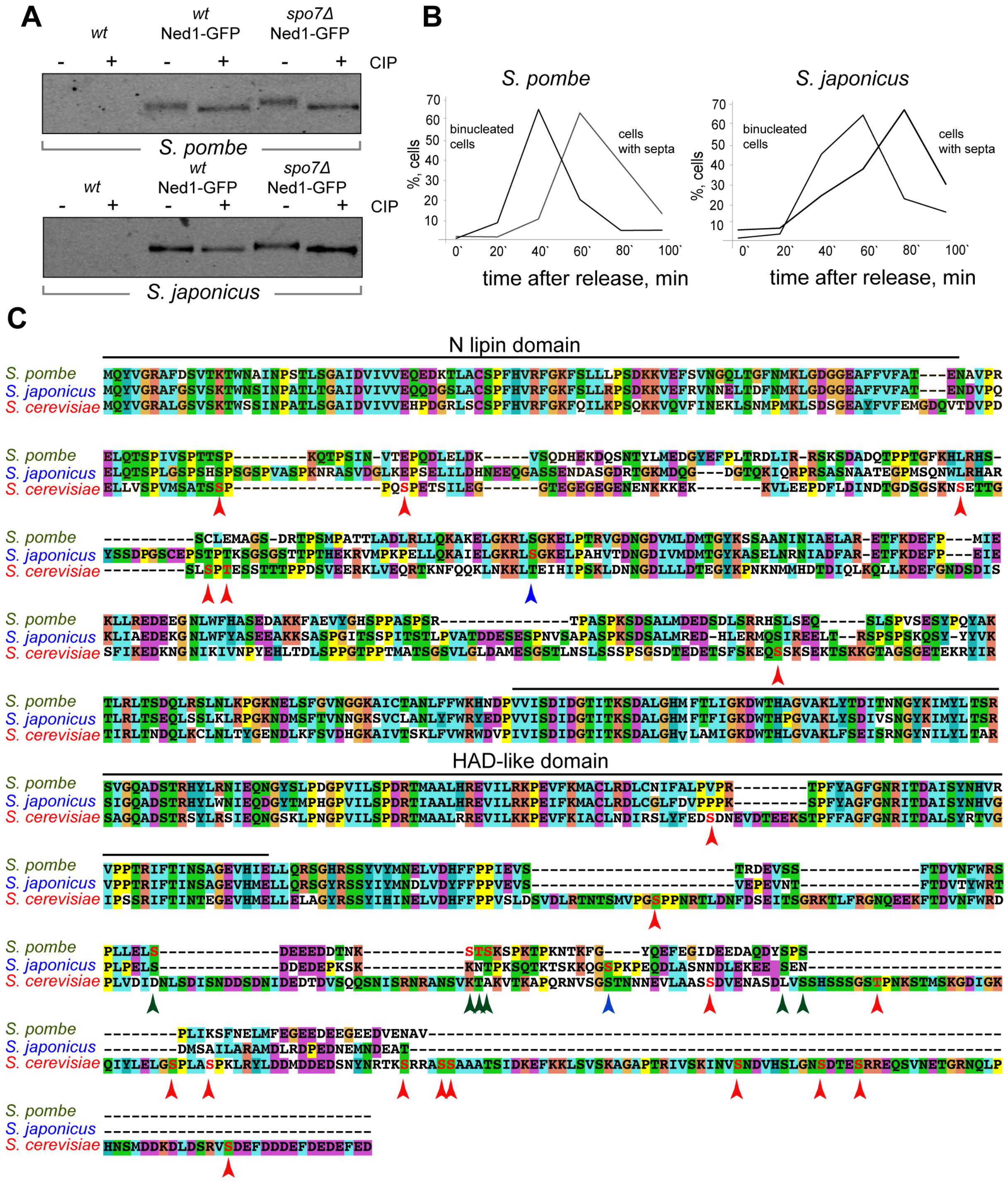

Makarova *et al.*, Fig. S3

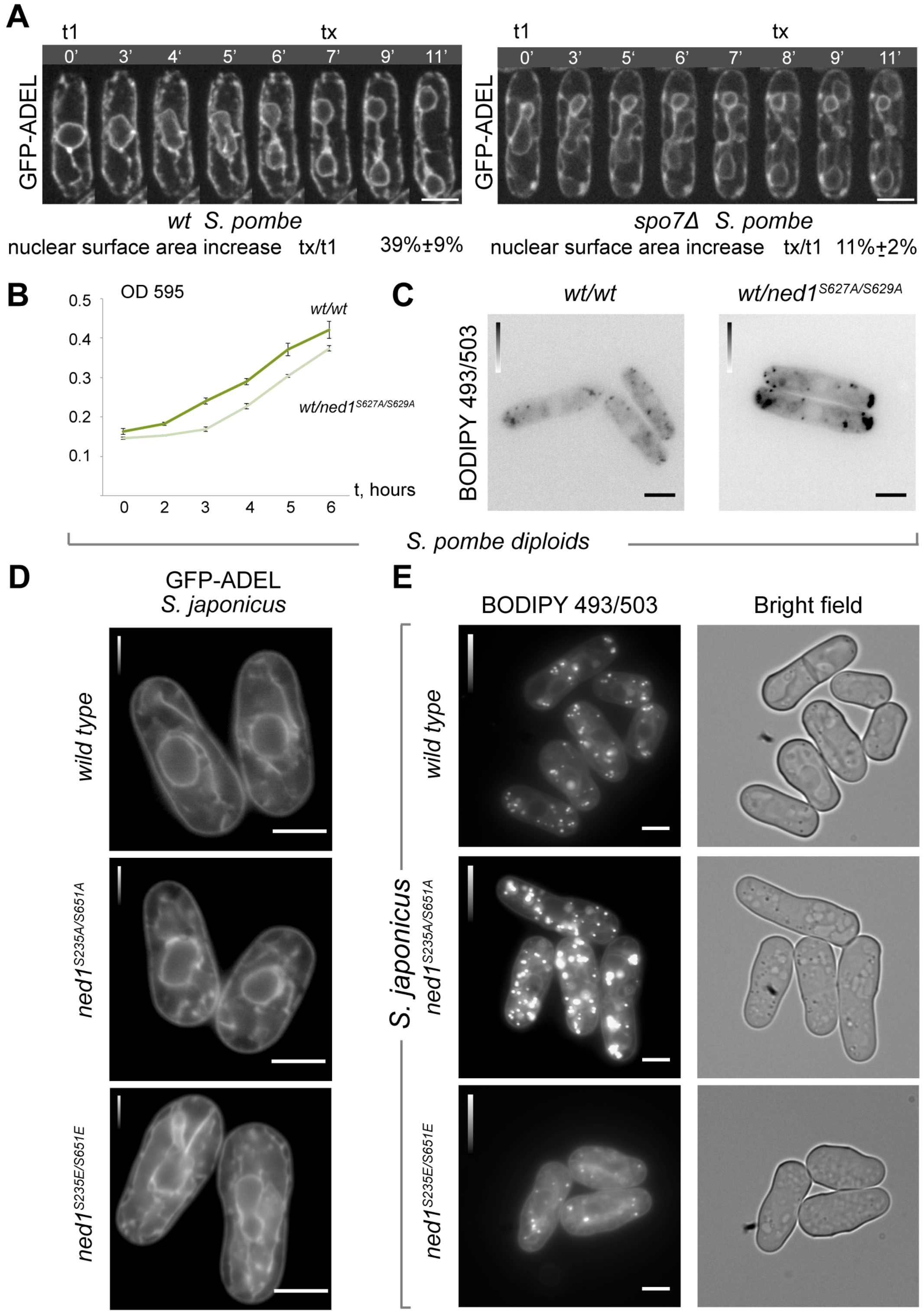

Makarova *et al.*, Fig. S4

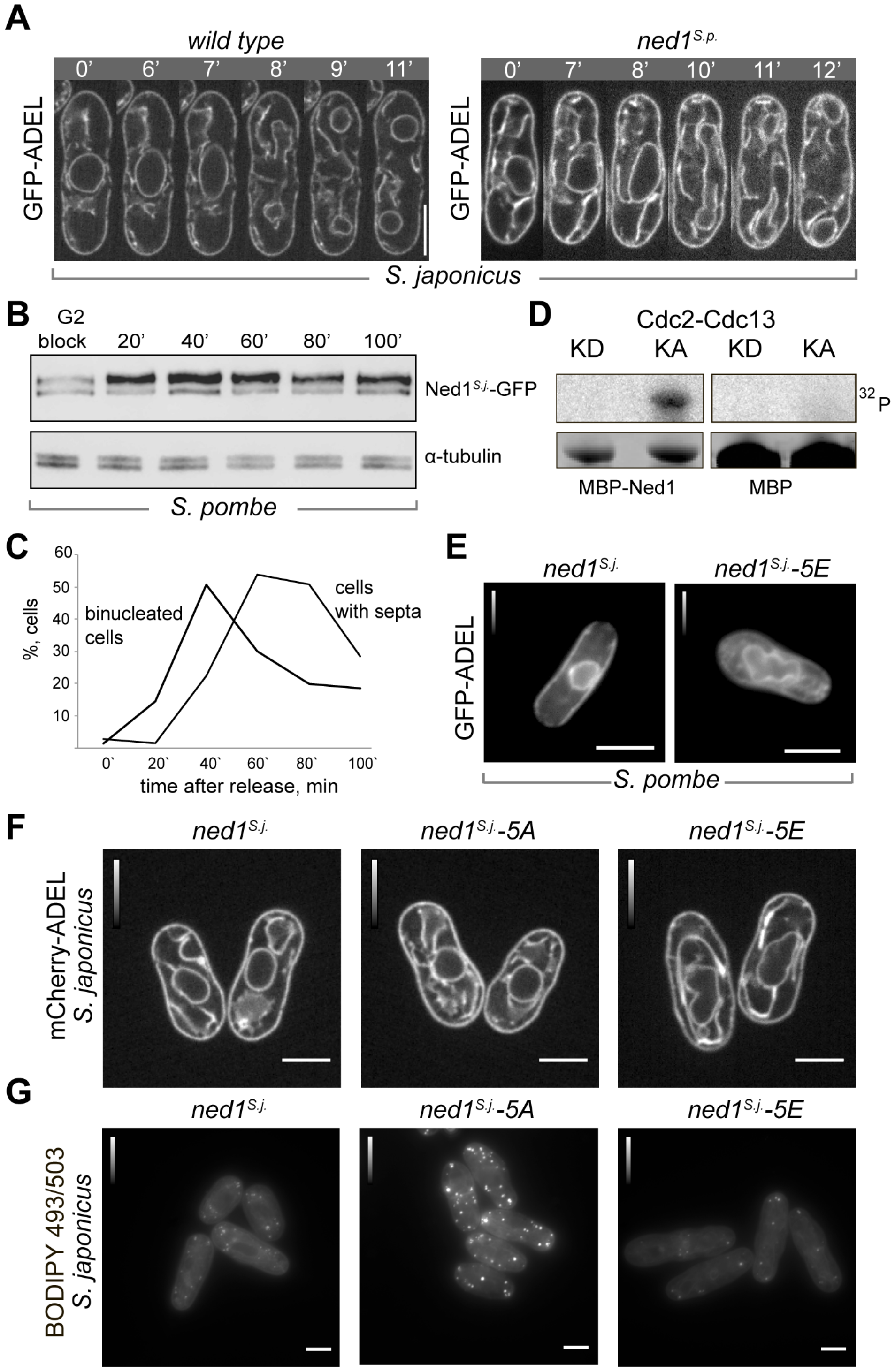

Supplemental Legends

Figure S1, related to Figure 1. (**A**) Inverted single z-plane epifluorescence images of *S. pombe* and *S. japonicus* cells of indicated genotypes expressing the nuclear pore marker Nup85-GFP. (**B**) Time-lapse sequence of GFP-ADEL (left) and Nhp6-mCherry (right) labeled *ned1Δ S. japonicus* cell undergoing mitosis. Note that the NE breaks down in anaphase similar to the control (compare with Fig. S4A). Time is in minutes. (**C**) Maximum projections of confocal z-stack images of *S. pombe* and *S. japonicus* cells of indicated genotypes co-expressing the nucleoplasmic marker Pus1-GFP and the mCherry-tagged nucleolar protein (Bop1 for *S. pombe* and Erb1 for *S. japonicus*). Note that NE extensions are not strictly associated with the nucleolus. **(D)** Single z-plane confocal images of *S. pombe* and *S. japonicus* cells of indicated genotypes expressing the luminal ER marker GFP-ADEL. $Ned1^{cat*}$ are catalytic mutants in both species. (**A**-**D**) Scale bars, 5μm. (**E**) Growth curves of *S. pombe* and *S. japonicus* cultures of indicated genotypes. Cells were grown at 30°C in the rich YES medium, with OD_{595} measurements performed each hour. Error bars represent standard deviation between three independent experiments. Unpaired t-test was used to obtain the p-values.

Figure S2, related to Figure 2. (A) Western blot (WB) analysis of immunoprecipitated Ned1-GFP from strains of indicated genotypes. CIP, treatment with alkaline calf intestinal phosphatase. (**B**) Exponentially growing *cdc25-22 S. pombe* and *cdc25-D9 S. japonicus* cells were blocked at the G2/M transition by incubating at 36°C for 3.5 hours. Cells were released into mitosis by cooling cultures to 24°C. Samples were taken at consecutive time points as indicated and fixed with paraformaldehyde. Septation index was counted in bright-field images; DAPI was used to evaluate the number of bi-nucleate anaphase cells (for each time point 400 cells were calculated). (**C**) Sequence alignment of the lipin proteins from *S. pombe*, *S. japonicus* and *S. cerevisiae*. Sequences were aligned using Clustal X ver. 2.1 [S1]. Assigned colors of the specific residues are based on alignment consensus. Arrows (*S. pombe*, green; *S. japonicus*, blue; *S. cerevisiae*, red) indicate phosphorylation sites identified by LC-MS/MS analyses. N-lipin and catalytic domains are indicated.

Figure S3, related to Figure 3. (A) Time-lapse sequences of the wild type (*left,* n=10) and *spo7Δ* (*right,* n=10) *S. pombe* cells undergoing mitosis. The ER is labeled with GFP-ADEL. Time is in minutes. Numbers indicate average experimentally determined values of the nuclear surface area increase between indicated time points. (**B**) Growth curves of diploid *wt/wt* and $wt/ned1^{S627AS629A}$ *S. pombe* cultures. Cells were grown at 30°C in the synthetic EMM medium, with OD_{595} measurements performed each hour. Error bars represent standard deviation between three independent experiments. (**C**) Maximum projection epifluorescence images of diploid *wt/wt* and $wt/ned1^{S627AS629A}$ *S. pombe* cells stained with the neutral lipid dye BODIPY 493/503. Shown are inverted images. (**D**) Single z-plane epifluorescence images of wild type, Ned1-S235A/S651A-GFP and Ned1-S235E/S651E-GFP *S. japonicus* cells expressing the ER marker GFP-ADEL. (**E**) Maximum projection epifluorescence images of wild type, Ned1-S235A/S651A-GFP and Ned1-S235E/S651E-GFP *S. japonicus* cells stained with the neutral lipid dye BODIPY 493/503. (**A**, **C**, **D**, **E**) Scale bars, 5 µm.

Figure S4, related to Figure 4. (**A**) Time-lapse sequences of mitotic *S. japonicus* cells co-expressing the ER marker GFP-ADEL together with the "wild type" Ned1-GFP (*top,* n=5) or its "transplanted" *S. pombe* ortholog (*Ned1$^{S.p.}$-GFP*) (*bottom,* n=4). (**B**) G2 block and cell cycle release of *cdc25-22* mutant *S. pombe* cells expressing Ned1$^{S.j.}$-GFP as an only source of Ned1. At each time point, Ned1$^{S.j.}$-GFP was immunoprecipitated and subjected to WB analysis. WB for α-tubulin present in input lysates was used as a loading control. (**C**) As a control for an experiment shown in (**B**), we provide quantification of the cell cycle block and release experiment. Exponentially growing *cdc25-22 S. pombe* cells expressing Ned1$^{S.j.}$-GFP were blocked at the G2/M transition by incubating at 36°C for 3.5 hours. Cells were released into mitosis by cooling cultures to 24°C. Samples were taken at consecutive time points as indicated and fixed with paraformaldehyde. Septation index was counted in brightfield images; DAPI was used to evaluate the number of bi-nucleate anaphase cells (for each time point 400 cells were calculated). (**D**) CDK1 phosphorylates the *S. japonicus* lipin Ned1 *in vitro*. Cdc2-Cdc13 kinase assays were performed using MBP-Ned1 purified from *E.coli*. MBP-Ned1 was incubated either with active (KA) or inactive (KD) Cdc2-Cdc13 kinase complexes. Kinase assays with MBP alone are shown as a control. (**E**) Single z-plane epifluorescence images of *S. pombe* cells co-expressing the ER marker GFP-ADEL and either the "wild type" or phosphomimetic mutant of Ned1$^{S.j.}$-5E-GFP as an only source of Ned1 protein. (**F**) Single z-plane confocal images of *S. japonicus* cells of indicated genotypes expressing ER marker mCherry-ADEL. (**G**) Maximum projection epifluorescence images of *S.*

japonicus cells of indicated genotypes stained with the neutral lipid dye BODIPY 493/503. (**A**, **E**, **F**, **G**) Scale bars, 5 μm.

Supplemental Table S1

S. pombe strains

Figure	Genotype	Collection No.
1B	*bip1-GFP-ADEL::ura4+ Nhp6-mCherry::ura4+ ade6-? leu1-32 ura4-D18*	SO7587
1B	*spo7Δ::ura4+ bip1-GFP-ADEL::ura4+ Nhp6-mCherry:ura4+ ade6-? leu1-32 ura4-D18*	SO7588
1B	*ned1 Δ:ura4+ bip1-GFP-ADEL::leu1+ Nhp6-mCherry:ura4+ ade6-? leu1-32 ura4-D18*	SO8006
1B	*nem1 Δ:ura4+ bip1-GFP-ADEL::leu1+ Nhp6-mCherry:ura4+ ade6-? leu1-32 ura4-D18*	SO7943
S1A	*spo7Δ::ura4+ nup85-GFP::ura4+ ade6-? leu1-32 ura4-D18*	SO7475
S1A	*nem1Δ:ura4+ nup85-GFP::ura4+ ade6-? leu1-32 ura4-D18*	SO7995
S1A	*ned1Δ:ura4+ nup85-GFP::ura4+ ade6-? leu1-32 ura4-D18*	SO7476
S1A	*nup85-GFP::ura4+ ade6-210 leu1-32 ura4-D18*	SO3985
S1C	*ned1Δ:ura4+ Pus1-GFP:ura4+ Bop1-mCherry:ura4+ ade6-? leu1-32 ura4-D18*	SO8105
S1C	*Pus1-GFP:ura4+ Bop1-mCherry:ura4+ ade6-? leu1-32 ura4-D18*	SO8100
S1D	*bip1-GFP-ADEL::leu1+ Ned1$^{D382E/D385E}$-GFP::ura4+ ade6-? leu1-32 ura4-D18*	SO7746
S1D, S3A	*bip1-GFP-ADEL::leu1+ ade6-? leu1-32 ura4-D18 h+*	SO4808
S1E	*ade6-210 leu1-32 ura4-D18 h+*	SO2865
S1E	*spo7Δ::ura4+ ade6-? leu1-32 ura4-D18 h+*	SO7472
S1E	*nem1Δ::ura4+ ade6-? leu1-32 ura4-D18 h+*	SO8002
S1E	*ned1Δ::ura4+ ade6-? leu1-32 ura4-D18 h+*	SO7909
S2A, 4E	*Ned1-GFP::ura4+ spo7Δ::ura4+ ade6-? leu1-32 ura4-D18*	SO7634
S2A, 2C, 2E, 4E	*Ned1-GFP::ura4+ ade6-? leu1-32 ura4-D18*	SO7644
2A, 2B, 2D, S2B	*Ned1-GFP::ura4+cdc25-22:ura4+ ade6-? leu1-32 ura4-D18*	SO7645
2E	*Ned1$^{S627E/S629E}$-GFP::ura4+ ade6-? leu1-32 ura4-D18*	SO7921
3A, 3B, 3C, S4D	*Ned1-GFP:ura4+ bip1-GFP-ADEL::leu1+ ade6-? leu1-32 ura4-D18*	SO7796
3A, 3B, 3C	*Ned1$^{S627E/S629E}$-GFP::ura4+ bip1-GFP-ADEL::leu1+ ade6-? leu1-32 ura4-D18*	SO7922
3D, S3B, 4A	Heterozygous diploid *Ned1-GFP::ura4+ ade6-210 ade216 leu1-32 ura4-D18*	SO7653
3D, 3E, S3B	Heterozygous diploid *Ned1$^{S627A/S629A}$-GFP::ura4+ ade6-210 ade216 leu1-32 ura4-D18*	SO7905
S3A	*spo7Δ::ura4+ bip1-GFP-ADEL::leu1+ ade6-? leu1-32 ura4-D18*	SO7643
4A, 4F	*Ned1-GFP $^{S.japonicus}$::ura4 ade6-? leu1-32 ura4-D18*	SO7747
4B	*bip1-mCherry-ADEL::leu1+ ade6-? leu1-32 ura4-D18*	SO5609
4B	*Ned1-GFP $^{S.japonicus}$::ura4+ bip1-mCherry-ADEL::leu1+ ade6-? leu1-32 ura4-D18*	SO7793
4D	Heterozygous diploid *Ned1$^{S.japonicus}$-GFP::ura4+ bip1-GFP-ADEL::leu1+ ade6-210 ade216 leu1-32 ura4-D18*	SO7985
4D	Heterozygous diploid *Ned1$^{S.japonicus}$-5A-GFP::ura4+ bip1-GFP-ADEL::leu1+ ade6-210 ade216 leu1-32 ura4-D18*	SO7986
S4D	*Ned1$^{S.japonicus}$-5E-GFP::ura4+ bip1-GFP-ADEL::leu1+ ade6-210 ade216 leu1-32 ura4-D18*	SO7989

4C, S4B, S4C	*Ned1$^{S.japonicus}$-GFP::ura4+ cdc25-22::ura4+ ade6-210 ade216 leu1-32 ura4-D18*	SO7791
4F	*Ned1-GFP $^{S.japonicus}$::ura4 ade6-? spo7Δ::ura4+ leu1-32 ura4-D18*	SO7898

S. japonicus strains

**Figure**	**Genotype**	**Collection No.**
1C	*bip1-GFP-ADEL::ura4+ Nhp6-mCherry::ura4+ ura4sj-D3 ade6sj-domE*	SOJ501
1C	*spo7Δ::ura4+ bip1-GFP-ADEL::ura4+ Nhp6-mCherry::ura4+ura4sj-D3 ade6sj-domE*	SOJ1506
1C, S1B	*ned1Δ:: kan^{R} bip1-GFP-ADEL::ura4+ Nhp6-mCherry::ura4+ ura4sj-D3 ade6sj-domE*	SOJ2197
1C	*nem1Δ::ura4+ bip1-GFP-ADEL::ura4+ Nhp6-mCherry::ura4+ura4sj-D3 ade6sj-domE*	SOJ2194
S1A	*Nup85-GFP::ura4+ura4sj-D3 ade6sj-domE*	SOJ54
S1A	*nem1Δ::ura4+ nup85-GFP::ura4+ ura4sj-D3 ade6sj-domE*	SOJ2193
S1A	*spo7Δ::ura4+ nup85-GFP::ura4+ ura4sj-D3 ade6sj-domE*	SOJ1504
S1A	*ned1Δ::kan^{R} nup85-GFP::ura4+ ura4sj-D3 ade6sj-domE*	SOJ1820
S1C	*Pus1-GFP:ura4+ Erb1-mCherry::ura4+ ura4sj-D3 ade6sj-domE*	SOJ2408
S1C	*ned1Δ::kan^{R} Pus1-GFP:ura4+ Erb1-mCherry::ura4+ ura4sj-D3 ade6sj-domE*	SOJ2483
S1D	*Ned1$^{D422E/D424E-}$GFP::ura4 bip1-GFP-ADEL::ura4+ ura4sj-D3 ade6sj-domE*	SOJ2012
S1D	*bip1-GFP-ADEL::ura4+ ura4sj-D3 ade6sj-domE*	SOJ495
S1E	*ade6sj-domE ura4sj-D3 h+*	SOJ88
S1E	*spo7Δ::ura4+ h+ ade6sj-domE ura4sj-D3*	SOJ1502
S1E	*ned1Δ::kan^{R} h+ ade6sj-domE ura4sj-D3*	SOJ1778
S1E	*nem1Δ::ura4+ h+ ade6sj-domE ura4sj-D3*	SOJ2191
2C, 4F, S2A, S3E, S4G	*Ned1-GFP::ura4+ h+ ade6sj-domE ura4sj-D3*	SOJ1410
4F, S2A	*Ned1-GFP::ura4+ spo7Δ::ura4+ ura4sj-D3 ade6sj-domE*	SOJ1879
2A, 2B, S2B	*Ned1-GFP::ura4+ cdc25-D9::ura4+ ura4sj-D3 ade6sj-domE*	SOJ1500
S3D,	*Ned1$^{S235A/S651A}$-GFP::ura4+ bip1-GFP-ADEL::ura4+ ura4sj-D3 ade6sj-domE*	SOJ2131
S3D,	*Ned1$^{S235E/S651E}$-GFP::ura4+ bip1-GFP-ADEL::ura4+ ura4sj-D3 ade6sj-domE*	SOJ2139
S3D, S4A, S4F	*Ned1-GFP::ura4+ bip1-GFP-ADEL::ura4+ ura4sj-D3 ade6sj-domE*	SOJ1453
S3E	*Ned1$^{S235A/S651A}$-GFP::ura4+ ura4sj-D3 ade6sj-domE*	SOJ2130
S3E	*Ned1$^{S235E/S651E}$-GFP::ura4+ ura4sj-D3 ade6sj-domE*	SOJ2137
S4E	*Ned1$^{S. pombe}$-GFP::ura4+ ura4sj-D3 ade6sj-domE*	SOJ2482
S4A	*Ned1$^{S. pombe}$-GFP::ura4+ bip1-GFP-ADEL::ura4+ ura4sj-D3 ade6sj-domE*	SOJ2486
S4F	*Ned1^{-5A}-GFP::ura4+ bip1-mCherry-ADEL::ura4+ ura4sj-D3 ade6sj-domE*	SO1793
S4F	*Ned1^{-5E}-GFP::ura4+ bip1-mCherry-ADEL::ura4+ ura4sj-D3 ade6sj-domE*	SO1799
S4G	*Ned1-^{5A}-GFP::ura4+ ura4sj-D3 ade6sj-domE*	SO1788
S4G	*Ned1-^{5E}-GFP::ura4+ ura4sj-D3 ade6sj-domE*	SO1790
S4E	*Ned1$^{S. pombe}$-GFP::ura4+ spo7Δ::ura4+ ura4sj-D3 ade6sj-domE*	SOJ2485

Supplemental Experimental Procedures

Strains and reagents

All strains used in this study are listed in Supplemental Table S1. *spo7Δ* and *nem1Δ* strains of both *S. japonicus* and *S. pombe* and the *ned1Δ S. pombe* mutant were constructed using the pJK210 plasmid backbone carrying the species-specific *ura4* cassettes flanked by 5' and 3' UTRs of respective genes. The *ned1Δ S. japonicus* strain was constructed using a standard PCR-based recombination method using KanMX6 as a selection marker. $Ned1^{D383E/D385E}$, $Ned1^{S627A/S629E}$, $Ned1^{S627E/S629E}$ mutants for *S. pombe* and $Ned1^{D422E/D424E}$, $Ned1^{S235A/S651A}$, $Ned1^{S235E/651E}$ for *S. japonicus* were generated by single end recombination using pJK210 backbone plasmids carrying the species-specific *ura4* cassettes. Replacement of *ned1* ORF in *S. pombe* with its *S. japonicus* ortholog was performed using a construct containing the 5'UTR and 3'UTR of $ned1^{S.pombe}$ that flanked the *ned1* $ORF^{S.japonicus}$. Replacement of *ned1* $ORF^{S.pombe}$ with mutant versions of $ned1^{S.japonicus}$ was performed similarly, with S499, S538, T620, T638 and S651 mutated to either alanine or glutamic acid. Replacement of *ned1* ORF in *S. japonicus* with its *S. pombe* ortholog was performed using a construct containing the 5'UTR and 3'UTR of $ned1^{S.japonicus}$ that flanked the *ned1* $ORF^{S.pombe}$. All Ned1 variants were tagged with GFP at C-terminus.

Please note that our data amend the automatic intron-exon annotation for the *S. japonicus ned1* gene provided by the Broad Institute [S2]. The second annotated 150bp-long intron is retained, resulting in insertion of additional 50 amino acids in a region between the N-lipin and HAD-like domains. D*e novo*

transcriptome assembly of RNA-seq data from both species confirmed that the second intron is retained in *S. japonicus* but not in *S. pombe* (data not shown). We did not observe any evidence for alternative splicing in the mitotic cycle. Sequencing of *ned1* cDNA isolated from *S. japonicus* confirmed the presence of the retained intron.

To create temperature sensitive mutants of *cdc25-D9*, a PCR-based random mutagenesis strategy was adapted from [S3] with some modifications. An adaptor strain that carries the C-terminally truncated *ura4* ORF cassette at the 3'UTR region of *cdc25* locus was generated using the plasmid pKanMX6-Ura4ΔC *S. japonicus*. Error-prone PCR was performed using the template plasmid pKS-Ura4ΔC *S. japonicus* that harbours *cdc25* ORF to be mutagenized. The adaptor strain was transformed with mutagenised PCR products and transformants were screened at 36°C for temperature-sensitive colonies.

Image acquisition and analysis

Time-lapse confocal images were obtained using Nikon Eclipse Ti-E inverted system equipped with CSU-X1 Spinning Disk Confocal fitted with Andor Ixon3 EM-CCD camera using 100x/1.4NA objective lens. Epifluorescence images were obtained with Zeiss Axio Observer Z1 microscope fitted with α Plan-FLUAR 100x/1.45NA objective lens and Hamamatsu Orca-Flash4.0 C11440 camera.

Nuclear circularity index was measured at the centre z-plane of interphase cells using formula $\Theta = 4\pi A/P^2 \sim 12.57A/P^2$, where Θ is

circularity, A is the area and P is the perimeter. Perimeter and area were measured using imageJ software (http://rsb.info.nih.gov/ij/; National Institutes of Health, Bethesda, MD, USA). Nuclear surface area in mitotic cells was measured as described previously [S4]. For interphase measurements we used late G2 cells (11-14 μm length).

Staining of lipid droplets in *S. japonicus* cells using BODIPY 493/503 was performed as in [S5]. Staining of membranes in *S. pombe* cells using DiO6 was performed as in [S6].

Growth rate measurements, cell cycle synchronization and germination of spores

Fission yeast cells grown overnight at 30°C to log-phase were diluted to OD_{595} 0.1. Optical density measurements were performed every hour until the OD_{595} reached approximately 0.6. All experiments were performed in three independent replicates.

S. pombe (*cdc25-22*) and *S. japonicus* (*cdc25-D9*) *cdc25* mutant cells grown overnight at the permissive temperature of 24°C in YES medium to OD_{595} 0.2-0.3 were shifted to 36°C for 3.5 hours. Release from the G2 block was performed by cooling cells in ice-cold water bath to 24°C followed by incubation at 24°C. Synchronization was verified by measuring the numbers of bi-nucleate and septated cells.

Sporulating heterozygous diploids were incubated with 0.5% glusulase (Perkin-Elmer) at 30°C for overnight. After washing with 30% ethanol cell

mixture was checked for the presence of vegetative cells. For selective germination washed spores were inoculated in minimal media lacking uracil and grown at 30°C for 24 hours.

Protein extraction and immunoprecipitation

Cell pellets were snap-frozen in liquid nitrogen and diluted in 100μl volume of IP buffer (50mM Tris-HCl, 150mM NaCl, 1% NP-40, 2 mM EDTA, 50 mM NaF, 0.1 mM Na3VO4, Roche protease inhibitor cocktail). Cell disruption was performed in IP buffer by homogenization with glass beads in a Mini Bead Beater (Biospec, Bartlesville, OK, USA) at 4°C. Total cell lysates were harvested and centrifuged at 16,000g for 10 minutes to remove cell debris. Soluble fractions were adjusted to the same total protein concentration, diluted with IP buffer excluding NP-40 and incubated with GFP-Trap beads (ChromoTek, Munich, Germany) for 2 hours at 4°C. Beads were washed 3 times with 1 ml of IP (excluding NP-40) and resuspended in SDS-loading buffer.

For phosphatase treatment experiments, proteins were extracted as above. Protein extracts were then incubated with GFP-trap beads, washed three times with buffer (50mM Tris-HCl, 150mM NaCl and Roche protease inhibitor cocktail) and subjected to phosphatase treatment on beads using either calf intestinal phosphatase (CIP; NEB) or the lambda phosphatase (λ, NEB) followed by resuspension in SDS-loading buffer.

Protein samples were subjected to SDS-PAGE and standard Western blotting. Ned1-GFP was probed by mouse *anti*-GFP antibody (Roche); mouse *anti-a*-tubulin antibody (kindly provided by K. Gould) served to monitor sample loading. For chemiluminescence detection we used FUSION Solo (secondary antibodies were anti-mouse-HRP (GE healthcare) followed by development with Clarity ECL substrate (Bio-rad). For some experiments, we used the Odyssey Infrared Imaging System (LI-COR Biosciences) with IRDye800 conjugated *anti*-mouse antibodies.

For Phos-tag assays, 6% acrylamide resolving gels containing 12,5 μM Phos-tag (Wako Pure Chemical Industries) and 12,5 μM $MnCl_2$ without SDS were prepared freshly for each experiment [S7]. To strengthen low percentage gels containing Phos-tag we increased amount of ammonium persulfate to 0.2%. Electrophoresis was carried out at constant current of 20mA for 3 hours. .

S. japonicus Ned1 ORF was cloned into pMal-c5x vector (NEB) for expression as a maltose binding protein (MBP) fusion. Protein expression was induced in *Escherichia coli* Rosetta2 strain overnight, using 0.4 mM IPTG at 18°C. Proteins were purified on amylose beads (NEB) according to the manufacturer's protocol.

Mass spectrometry (MS) analysis

Isolated proteins were denatured, reduced with Tris 2-carboxyethyl phosphine, alkylated with iodoacetamide, and digested overnight at 37˚C with

Trypsin Gold (Promega) or Chymotrypsin (Princeton Separations) after diluting to 2M urea with 50 mM Tris pH 8.5. The resulting peptides were subjected to 2D LC-MS/MS (MudPIT) on a Thermo LTQ as previously detailed [S8]. Thermo RAW files were converted to DTA files using Scansifter [S9] and spectra with fewer than 20 peaks were excluded from analysis. Spectra were searched against the *S. pombe* database (pombase.org, May 2011) using the SEQUEST (TurboSequest v.27 rev12) algorithm on a high performance computing cluster (Advanced Computing Center for Research & Education at Vanderbilt University). We added contaminant proteins (e.g. keratin, IgG) to the complete *S. pombe* database and reversed and concatenated all sequences to allow estimation of false discovery rates (10186 total entries). Variable modifications (C+57, M+16, [STY]+80 for all spectra), strict trypsin cleavage, <10 missed cleavages, fragment mass tolerance: 0.00 Da (because of rounding in SEQUEST, this results in 0.5 Da tolerance), and parent mass tolerance: 2.5 Da were allowed. Peptide identifications were assembled and filtered in Scaffold (v4.1.1, Proteome Software, Portland, OR) using the following criteria: minimum of 99% protein identification probability; minimum of 5 unique peptides; minimum of 90% peptide identification probability; minimum peptide length of five amino acids; minimum number of one tryptic terminus. Scaffold PTM was used to filter the spectra and analyze phosphorylation sites (v2.1.3, Proteome Software, Portland, OR). Phosphorylation sites are reported with their site localization scores and Ascores [S10].

***In vitro* kinase assay**

Immunoprecipitated wild type or mutated Ned1-GFP was washed three times with protein kinase buffer (10 mM Tris, pH 7.4, 10 mM MgCl2, and 1 mM DTT), and then resuspended in kinase buffer supplemented with 10 µM cold ATP, 5 µCi of [^{32}P]ATP, and 100 ng of purified insect cell–produced Cdc2-Cdc13 in a total volume of 20 µl. The reactions were incubated at 30°C for 30 minutes before being terminated by the addition of SDS sample buffer. Proteins were separated by 4-12% NuPAGE Bis-Tris Precast Gels (Life Technologies, Grand Island, NY), transferred to polyvinylidene fluoride (PVDF) membrane, and phosphorylated proteins were visualized by autoradiography. The same membrane was wetted with methanol and blotted with anti-GFP antibody (Roche, Indianapolis, IN) to visualize the level of proteins used in the kinase assays. MBP-Ned1$^{S.\ japonicus}$ and MBP proteins were incubated with Cdc2-Cdc13 complexes and separated on SDS-PAGE, followed by staining with Coomassie blue G250 to visualize the proteins. The protein gel was then dried in a gel dryer, followed by exposure to film to examine phosphorylation.

Supplemental References

S1. Larkin, M.A., Blackshields, G., Brown, N.P., Chenna, R., McGettigan, P.A., McWilliam, H., Valentin, F., Wallace, I.M., Wilm, A., Lopez, R., et al. (2007). Clustal W and Clustal X version 2.0. Bioinformatics *23*, 2947-2948.

S2. Rhind, N., Chen, Z., Yassour, M., Thompson, D.A., Haas, B.J., Habib, N., Wapinski, I., Roy, S., Lin, M.F., Heiman, D.I., et al. (2011). Comparative functional genomics of the fission yeasts. Science *332*, 930-936.

S3. Tang, X., Huang, J., Padmanabhan, A., Bakka, K., Bao, Y., Tan, B.Y., Cande, W.Z., and Balasubramanian, M.K. (2011). Marker reconstitution mutagenesis: a simple and efficient reverse genetic approach. Yeast *28*, 205-212.

S4. Yam, C., He, Y., Zhang, D., Chiam, K.H., and Oliferenko, S. (2011). Divergent strategies for controlling the nuclear membrane satisfy geometric constraints during nuclear division. Current biology : CB *21*, 1314-1319.

S5. He, Y., Yam, C., Pomraning, K., Chin, J.S., Yew, J.Y., Freitag, M., and Oliferenko, S. (2014). Increase in cellular triacylglycerol content and emergence of large ER-associated lipid droplets in the absence of CDP-DG synthase function. Molecular biology of the cell *25*, 4083-4095.

S6. Koning, A.J., Lum, P.Y., Williams, J.M., and Wright, R. (1993). DiOC6 staining reveals organelle structure and dynamics in living yeast cells. Cell motility and the cytoskeleton *25*, 111-128.

S7. Kinoshita, E., Kinoshita-Kikuta, E., Takiyama, K., and Koike, T. (2006). Phosphate-binding tag, a new tool to visualize phosphorylated proteins. Molecular & cellular proteomics : MCP *5*, 749-757.

S8. Roberts-Galbraith, R.H., Chen, J.S., Wang, J., and Gould, K.L. (2009). The SH3 domains of two PCH family members cooperate in assembly of the Schizosaccharomyces pombe contractile ring. The Journal of cell biology *184*, 113-127.

S9. Ma, Z.Q., Tabb, D.L., Burden, J., Chambers, M.C., Cox, M.B., Cantrell, M.J., Ham, A.J., Litton, M.D., Oreto, M.R., Schultz, W.C., et al. (2011). Supporting tool suite for production proteomics. Bioinformatics *27*, 3214-3215.

S10. Beausoleil, S.A., Villen, J., Gerber, S.A., Rush, J., and Gygi, S.P. (2006). A probability-based approach for high-throughput protein phosphorylation analysis and site localization. Nature biotechnology *24*, 1285-1292.
